# Genomic divergence between *Dickeya zeae* strain EC2 isolated from rice and previously identified strains, suggests a different rice foot rot strain

**DOI:** 10.1371/journal.pone.0240908

**Published:** 2020-10-20

**Authors:** Jingxin Zhang, Mohammad Arif, Huifang Shen, John Hu, Dayuan Sun, Xiaoming Pu, Qiyun Yang, Birun Lin

**Affiliations:** 1 Key Laboratory of New Technique for Plant Protection in Guangdong, Institute of Plant Protection, Guangdong Academy of Agricultural Sciences, Guangzhou, China; 2 Department of Plant and Environmental Protection Sciences, College of Tropical Agriculture and Human Resources, University of Hawaii, Honolulu, HI, United States of America; ICAR-Indian Institute of Wheat and Barley Research, INDIA

## Abstract

Rice foot rot caused by *Dickeya zeae* is an important bacterial disease of rice worldwide. In this study, we identified a new strain EC2 from rice in Guangdong province, China. This strain differed from the previously identified strain from rice in its biochemical characteristics, pathogenicity, and genomic constituents. To explore genomic discrepancies between EC2 and previously identified strains from rice, a complete genome sequence of EC2 was obtained and used for comparative genomic analyses. The complete genome sequence of EC2 is 4,575,125 bp in length. EC2 was phylogenetically closest to previously identified *Dickeya* strains from rice, but not within their subgroup. In terms of secretion systems, genomic comparisons revealed that EC2 harbored only type I (T1SS), typeⅡ (T2SS), and type VI (T6SS) secretion systems. The flagella cluster of this strain possessed specific genomic characteristics like other *D*. *zeae* strains from Guangdong and from rice; within this locus, the genetic diversity among strains from rice was much lower than that of within strains from non-rice hosts. Unlike other strains from rice, EC2 lost the zeamine cluster, but retained the clustered regularly interspaced short palindromic repeats-1 (CRISPR-1) array. Compared to the other *D*. *zeae* strains containing both exopolysaccharide (EPS) and capsular polysaccharide (CPS) clusters, EC2 harbored only the CPS cluster, while the other strains from rice carried only the EPS cluster. Furthermore, we found strain MS1 from banana, carrying both EPS and CPS clusters, produced significantly more EPS than the strains from rice, and exhibited different biofilm-associated phenotypes. Comparative genomics analyses suggest EC2 likely evolved through a pathway different from the other *D*. *zeae* strains from rice, producing a new type of rice foot rot pathogen. These findings emphasize the emergence of a new type of *D*. *zeae* strain causing rice foot rot, an essential step in the early prevention of this rice bacterial disease.

## Introduction

Rice foot rot is an important bacterial disease affecting rice (*Oryza sativa* L.), and first reported in Japan [1], and spread to the other important rice-growing countries, including China, South Korea, the Philippines, India, Indonesia, and Bangladesh [2–4]. It has become one of the most important bacterial diseases of rice in India [5]. In China, the disease has caused significant losses in Zhejiang, Shanghai, Jiangsu, Hubei, and Fujian provinces before 1983 [6]. Currently, it is omnipresent in most of the major rice-growing areas in China, and has become an important threat to rice production [4]. Therefore, the epidemiologic mechanisms of this disease, and the pathogenesis of the causal pathogen have raised extensive concerns.

The causal agent of rice foot rot disease is *Dickeya zeae* (previously *Erwinia chrysanthemi* pv. *zeae*), family *Pectobacteriaceae*. The genus includes: *D*. *zeae*, *D*. *chrysanthemi*, *D*. *dianthicola*, *D*. *paradisiaca*, *D*. *dadantii* subsp. *dadantii*, *D*. *dadantii* subsp. *dieffenbachiae*, *D*. *solani*, *D*. *aquatica*, *D*. *fangzhongdai*, *D*. *undicola*, and *D*. *lacustris* [7–14]. *D*. *zeae* contains a group of pathogens infecting important plants in the Poaceae like rice and maize, which indicate distinct genetic variants in *D*. *zeae* [15, 16]. This was consistent with the genetic evolution analysis of *Dickeya* species based on *recA* gene sequences; indicated that there were different sequence variants (sequevars) isolated from different host plants existed among *D*. *zeae* strains [17]. Moreover, no common antigens among *D*. *zeae* strains from rice or maize were found in serological tests. The strains from rice, however, exhibited higher pathogenicity and broader host range than strains from maize [18]. These findings indicate the differentiation and genetic evolution of *D*. *zeae* strains might be an outcome of long-term interactions between pathogens and hosts.

Until now, there were 11 complete or draft genomes of *D*. *zeae* deposited at the National Center for Biotechnology Information (NCBI) GenBank Genome database. Among them, three strains were from rice, including EC1 (complete, Guangdong, China), ZJU1202 (draft, Guangdong, China) and DZ2Q (draft, Italy); in a phylogenetic analyses, all three strains from rice were grouped in a clade separated from the other *D*. *zeae* strains [15]. The *D*. *zeae* strains from rice may fall in a distinct group, as they showed some unique genomic features, like the zeamine phytotoxins biosynthesis gene cluster [15], and incomplete subtype I-F CRISPR array. In this study, however, we isolated a new *D*. *zeae* strain from rice in Guangdong, China, with obvious differences in cultural and biochemical characteristics, and genomic determinants from the typical strain EC1 from rice. Previous studies on the virulence factors of *D*. *zeae* strains from rice focused on the acyl homoserine-lactone regulated quorum-sensing system [3], zeamine biosynthesis gene cluster [19–21], zeamine biosynthesis-related transcription factors SlyA [22] and Fis [23], as well as the twin-arginine translocation system [24]. These genomic traits might not reflect the comprehensive strain differentiation and genetic evolution of the identified strains from rice. In this study, therefore, we characterized the complete genome sequence of a newly identified strain *D*. *zeae* EC2 isolated from diseased rice plant and compared with previously identified strains from rice, EC1, ZJU1202, and DZ2Q, and other *D*. *zeae* strains. We also analyzed the differences in some cultural and pathogenicity characteristics among the newly identified strain from rice, *D*. *zeae* EC2, the previously identified strain, *D*. *zeae* EC1, and the strain from banana, *D*. *zeae* MS1. The genome-wide comparison and culture and pathogenicity would help to clarify the differentiation among *D*. *zeae* strains from rice and their genetic adaptation to the host. This information might raise more attention to this newly emerging rice pathogen and provide better insight into the early prevention of rice foot rot disease.

## Materials and methods

### Bacterial strains and culture media

Three rice plants with typical symptoms of rice foot rot were collected from the fields of Qingyuan city, Guangdong province, China. The margins between diseased and healthy areas at the base of stems were cut into pieces, surface-sterilized in 75% ethanol for 30 s and 1% NaOCl for 1 min, and then rinsed three times with sterile water. The pieces were macerated in 100 μL of sterile water, and the mixture were streaked onto nutrient agar (NA) plates (3 g/L beef extract, 0.5 g/L yeast extract paste, 5 g/L peptone, and 10 g/L agar, pH 7.0) and incubated for 48 h at 30°C. Single colonies were picked and subcultured three times; one typical strain, EC2, was confirmed through Koch's postulates and identity was confirmed using 16S rDNA primers [25] and *recA* [26]. South China Agricultural University provided *D*. *zeae* EC1 strain, while our group isolated MS1 and CE1 (*Canna edulis* strain) strains. These strains were stored in glycerol at -80°C at the Key Laboratory of New Technique for Plant Protection in Guangdong province, China. For subculture, bacteria were grown to a concentration of 10^8^ CFU/mL at 30°C in Luria-Bertani (LB) broth (10 g/L bacto tryptone, 5 g/L yeast extract, and 10 g/L NaCl, pH 7.0) in an incubator shaker at 100 rpm.

### Biolog analysis

*Dickeya zeae* strains, EC1, EC2, MS1 and CE1, were characterized using GEN III MicroStation (Biolog, Hayward, CA, USA). A 150-μL bacterial suspension of 10^8^ CFU/mL was added to each well of a GEN III micro-plate (Biolog) containing 95 sugars, alcohols, acids, amines, and other substrates. Culture suspensions were incubated and read, and then compared to the databases using the automated microbial analysis system, Biolog.

### Pathogenicity test

Bacteria were grown to a concentration of 10^8^ CFU/mL in LB broth, and diluted fivefold. A 200-μL bacterial dilution of each strain EC1 and EC2 was injected into the basal stem of each rice seedling at the tillering stage. Rice seedlings inoculated with sterile water served as a negative control. The rice seeds were provided by the College of Agriculture, South China Agricultural University, China, and were grown in the greenhouse of our lab. The injection sites were wrapped in cotton balls moisturized with sterile water. The inoculated plants were placed in the greenhouse at 30°C and 90% relative humidity. Seedlings with soft rot symptoms were used for bacterial isolation, and re-isolated strains were confirmed by 16S rDNA amplification and sequencing.

Bacteria were grown to a concentration of 10^9^ CFU/mL in LB broth. Half of the culture was kept as culture crude, and the remaining half was centrifuged for 10 min at 12,000 rpm; the supernatant pipetted as culture extract. The non-inoculated LB broth was used as a negative control. Each of the 20 rice seeds was immersed in 20 mL of prepared culture crude or culture extract for 6 h at room temperature. The treated seeds were washed three times in sterilized water and transferred onto moistened filter papers in petri dishes. The seeds were incubated for one week at 28°C and 14-h-light and 10-h-dark conditions.

### Genomic DNA extraction and genome sequencing

Total DNA was extracted from 2-mL bacterial suspensions (10^8^ CFU/mL) using the TIANamp Bacterial DNA Kit (Tiangen Biotech, Beijing, China) according to manufacturer's directions. The fragmented genomic DNAs were treated with G-tubes (Covaris) and end-reparation. The treated DNAs were used to construct a SMRTbell DNA library with fragment sizes of >10 Kb. Next, SMRT sequencing was conducted using the Pacific Biosciences RSII sequencer (Pacific Biosciences, Menlo Park, United States) at Genedenovo (Guangzhou, China), according to standard protocols using P4-C2 chemistry. Reads (≥500 bp) with a quality value of over 0.75 were processed for next step. A Hierarchical genome-assembly process pipeline [27] was used to correct random errors in long seed reads (≥ 6 Kb) by aligning shorter reads against them. A total of 115,312 reads with an average length of 9,047 bp and totaling 1,043,332,658 bp, were obtained, and *de novo* assembled using the Celera assembler with overlap-layout-consensus strategy [28]. The Quiver consensus algorithm [27] was used to validate the quality of the assembled genome sequence. A circularized genome of 4,574,986 bp with 53.34% GC content was generated after trimming the ends of the assembled sequence. To correct this assembly, another DNA library was constructed for next-generation sequencing. The paired-end library was sequenced on an Illumina HiSeq 4000 (Illumina, San Diego, CA, USA) at Genedenovo (Guangzhou, China). A total of 14,457,752 output reads were aligned to the assembly of PacBio sequencing using a Burrows-Wheeler alignment tool with default parameters [29], and 14,255,069 of them were matched to the circularized genome. Pilon was also used to improve the genome sequence by searching for inconsistencies between the genome sequence and the reads [30]. In terms of insertions, deletions, and substitutions, 170, 31, and 1 correction were made, respectively, to the circularized genome. The closed genome was corrected to 4,575,125 bp in length with a GC content of 53.34%.

### Annotation of the *D*. *zeae* EC2 genome

RepeatMasker [31], rRNAmmer [32], and tNRAscan [33] were applied to search for repetitive elements, noncoding RNAs, and tRNAs, respectively, and gene finding was performed with the GeneMarkS program [34]. Next, we annotated the functions of the predicted genes through similarity searches against several databases: NCBI Non-redundant Protein Database (ftp://ftp.ncbi.nih.gov/blast/db/FASTA/nr.gz), UniProt/Swiss-Prot (http://www.uniprot.org/downloads), Kyoto Encyclopedia of Genes and Genomes (http://www.genome.jp/kegg/), Gene Ontology, Cluster of orthologous groups of proteins (COG), and Protein Families (ftp://ftp.ebi.ac.uk/pub/databases/Pfam). GC contents, GC-skew value (GC skew = [G-C]/[G+C]), tRNA, rRNA, predicted genes in positive and negative strands, and COG annotation, were presented using Circos [35]. We also used CRISPRFinder [36] and antiSMASH 4.0 [37] to predict CRISPRs and gene clusters of polyketides (PKs) and nonribosomal peptides (NRPs), respectively.

### Phylogenetic analysis of *Dickeya* strains

Twenty-three *Dickeya* strains with complete or draft genome sequences were used to extract the gene sequences of *dnaX*, *recA*, *dnaN*, *fusA*, *gapA*, *purA*, *rplB*, *rpoS*, and *gyrA* [11], plus an out-group control, *Pectobacterium carotovorum* subsp. *carotovorum* PC1. The concatenated sequences of these nine genes were processed on MEGA 5.1 using a neighbor-joining algorithm with 1,000 bootstrapped replications. To obtain an estimate of overall genomic similarities, the genome sequences of 13 *D*. *zeae* strains were processed to calculate average nucleotide identity (ANI) and alignment percentage (AP) using CLC Genomics Workbench 12.0.3 (Qiagen, USA), and *in silico* DNA-DNA hybridization (isDDH) [38] by adapting formula 2.

### Genomic comparison of available genomes of *D*. *zeae* strains or strains from rice

Genomic annotation of 13 whole-genome sequences of *D*. *zeae* strains and one well-sequenced strain, *D*. *dadantii* 3937, were retrieved from NCBI and used for genomic comparison. Synteny analysis was performed on Mummer (http://mummer.sourceforge.net/) by conducting nucleotide-nucleotide sequence comparisons. The alignments of pairs of nucleic acid sequences were marked at their coordinate positions in the size-reduced synteny graphs. To determine the orthologous relationships of coding proteins for *D*. *zeae* strains, orthoMCL [39] was used to identify the set of common genes or specific genes through a BLAST search with the following parameters: p-value cut-off = 1 × 10^−5^; identity cut-off = 80%.

### Assays of exopolysaccharide (EPS) production

Single colonies of strains EC2, EC1, and MS1, were transferred to 15 mL nutrient broth (NB; 3 g/L beef extract, 0.5 g/L yeast extract paste, and 5 g/L peptone, pH 7.0) and incubated at 28°C until OD600 1.0. For colony morphology assay, one-μL bacteria culture was spotted onto NA plates supplemented with 2% glucose, and sub-cultured for 4 d at 28°C [40]. EPS production was measured as follow: three-mL bacteria culture was transferred into 300 mL NB medium, and grown for 5 d at 200 rpm and 28°C. The culture was centrifuged for 30 min at 10,000 rpm, and 250 mL of supernatant collected; 20% KC1 (w/v) was added to make a final KC1 concentration of 1%. After this, a double volume of ethanol was added to this solution; the mixture was placed at 4°C for 24 h followed by centrifugation for 30 min at 10,000 rpm. The pellet was dried at 55°C and measured the weight [41]. The experiment was repeated in triplicate, and two-tailed t-tests were performed to evaluate the differences.

## Results

### The *D*. *zeae* pathogen was found to cause rice foot rot in Qingyuan city, China

**Fig 1:**
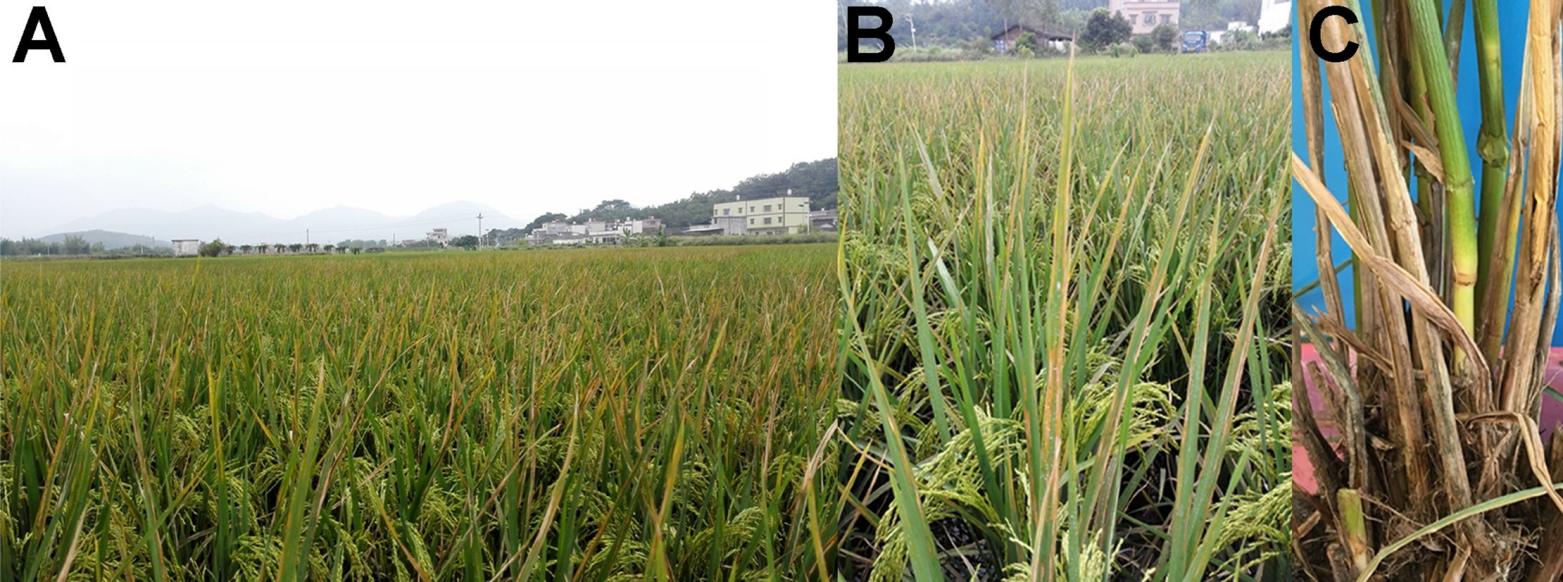
Symptoms of rice foot rot observed in the fields of Qingyuan, Guangdong province, China. (A) Yellowing and necrosis of rice leaves. (B) Rice spikes fail to pollinate and form grains and leaves are yellow and necrotic. (C) The crowns of diseased rice stems turn dark and rot.

**Fig 2:**
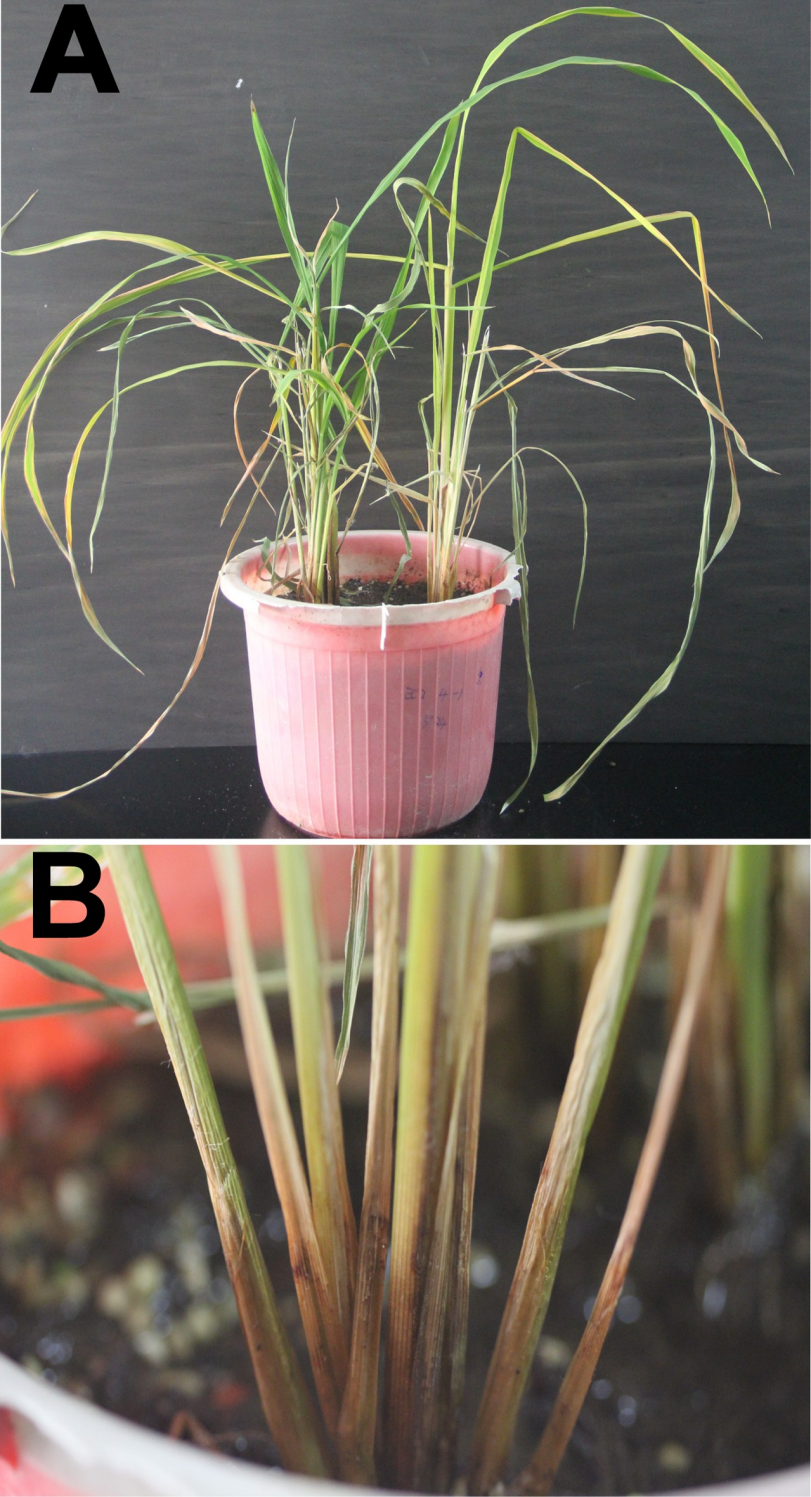
Typical rice foot rot symptoms observed after inoculation with new strain, EC2, isolated from rice. (A) New leaves are wilting and older leaves are yellowing 11 d after inoculation with strain EC2. (B) The base of rice stems are dark and rotten 11 d after inoculation with strain EC2.

In October 2016, rice plants from the fields of Qingyuan city showed symptoms of leaf yellowing and necrosis (Fig 1A), and rice spikes failed to pollinate and form grains (Fig 1B). The rice stems turned dark with soft rot near the soil line, and were easily broken (Fig 1C). Typical strain EC2 was isolated from the diseased rice plant. Pathogenicity tests showed that the new strain isolated from rice can cause typical foot rot symptoms as previously verified for strain EC1 that was also isolated from rice in Guangdong. These symptoms included shrinking and yellowing of newly emerging and older leaves, respectively (Fig 2A); soft rot at the foot of stems (Fig 2B). Biolog analysis confirmed EC2 as the strain from genus *Dickeya*, as well as other *D*. *zeae* strains from Guangdong Province (EC1 isolated from rice, MS1 isolated from banana, and CE1 isolated from *C*. *edulis* ker.). Principal coordinates analysis revealed that this new strain EC2 was much different from previously identified strain from rice, EC1, in the use of different substrates as well as other *D*. *zeae* Guangdong strains (S1 Fig).

### Genome assembly and annotation

**Fig 3:**
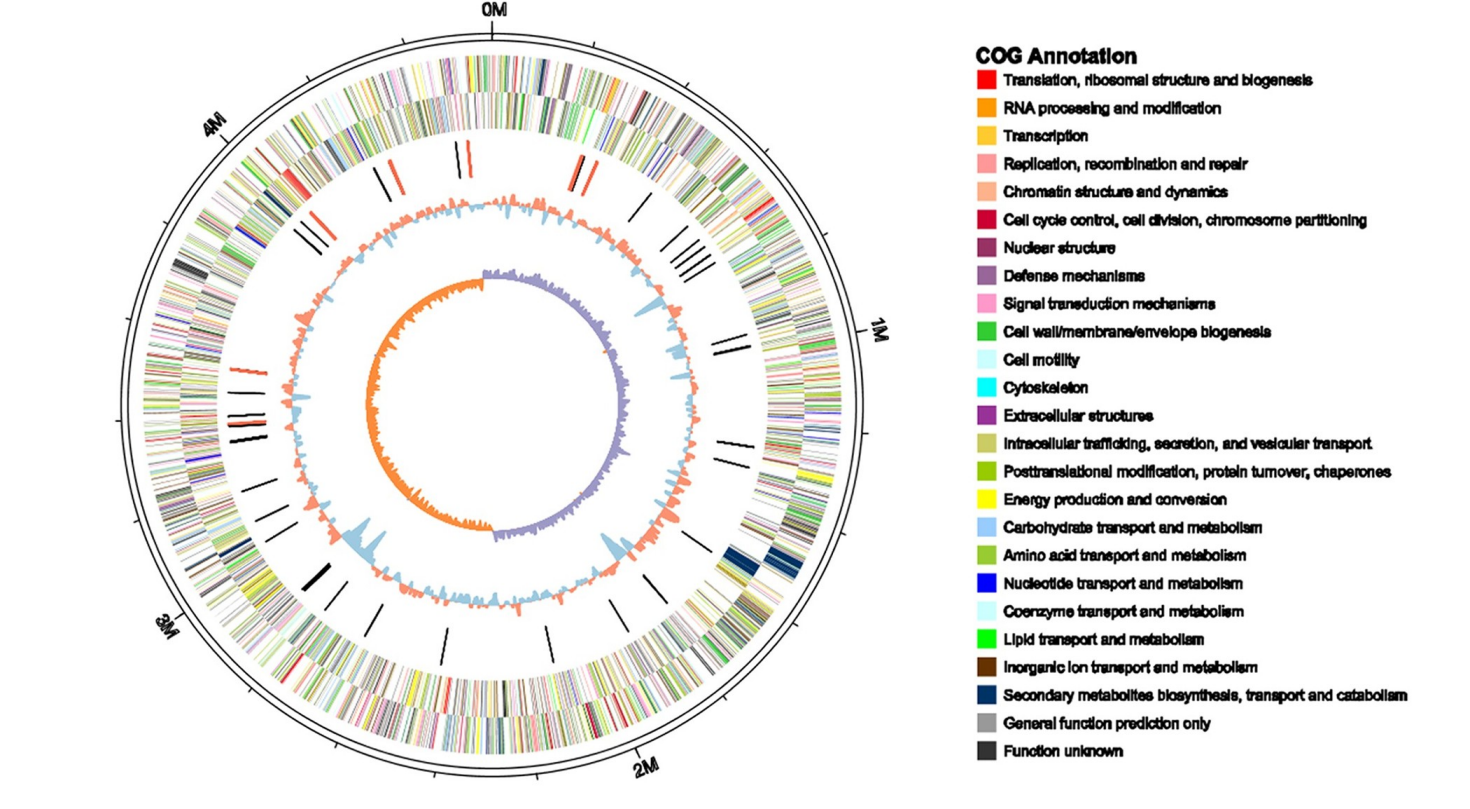
Circular visualization of the complete genome of *D*. *zeae* EC2. From outside to inside, the circles indicate predicted genes on the positive strand, predicted genes on the negative strand, ncRNAs (black indicates tRNAs, red indicates rRNAs), G+C contents and GC skew values (GC skew = (G-C)/(G+C); purple indicates >0, orange indicates <0), respectively.

The complete genome of strain EC2 was 4,575,125 bp in length with a 53.34% GC content, and predicted 3,999 coding sequences. Information on predicted gene distribution, the COG annotation, and GC content was represented on the circular chromosome beginning at the *dnaA* start codon (Fig 3). In the genome, we found 75 tRNA and 7 rRNA regions. Within the predicted rRNA regions, two and four common organization types (16S-23S-5S) were predicted on the positive and negative strands, respectively, while an unusual organization type (16S-23S-5S-5S) was predicted on the negative strand. The complete genome project of EC2 was deposited under NCBI GenBank accession no. CP031515.

### Phylogenetic analysis of *Dickeya* strains

**Fig 4:**
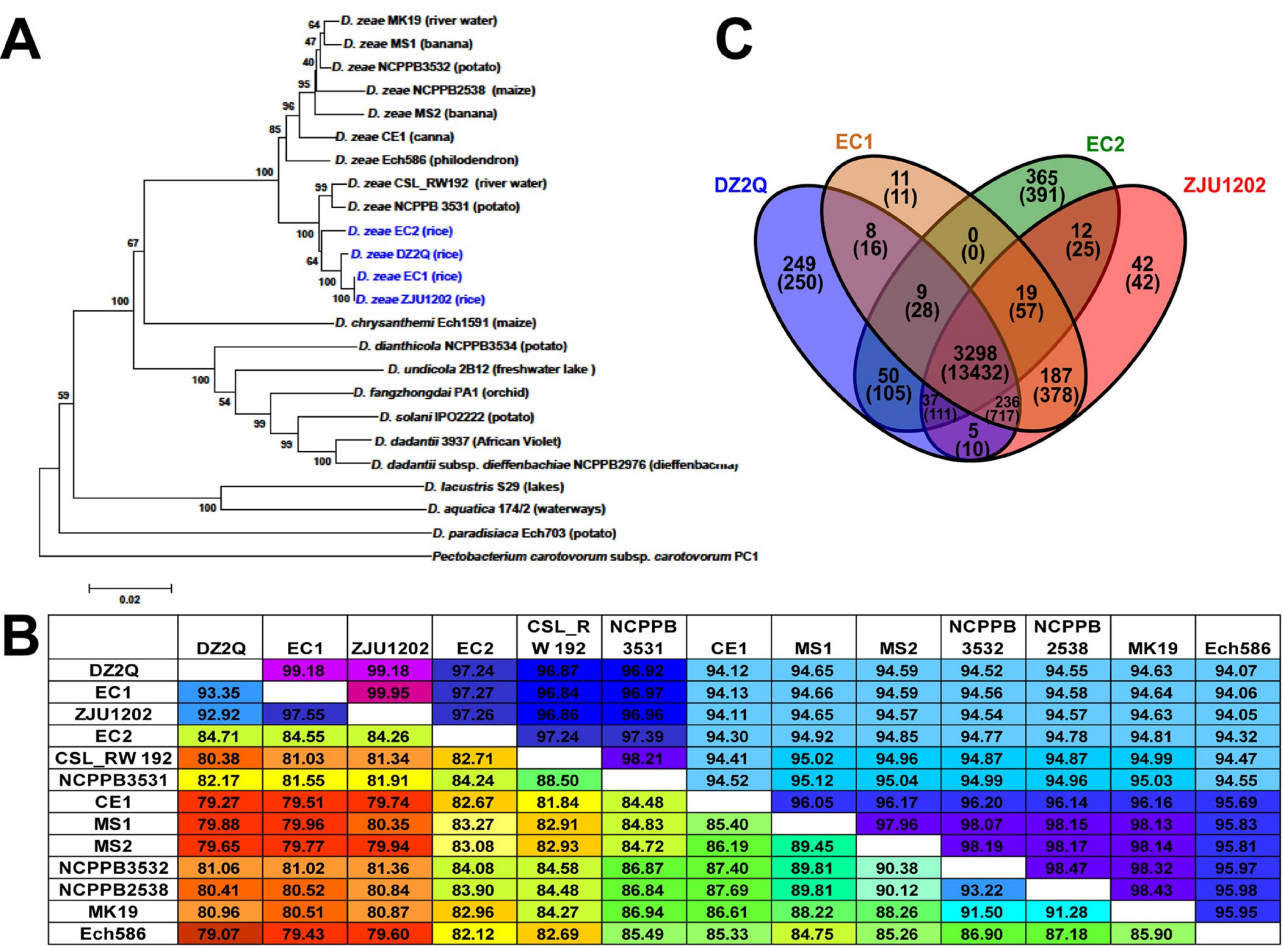
Phylogenetic placement of *D*. *zeae* strain EC2 and its common or specific genes with other strains isolated from rice. (A) Phylogenetic analysis of *Dickeya* strains based on concatenated sequences of the genes, *dnaX*, *recA*, *dnaN*, *fusA*, *gapA*, *purA*, *rplB*, *rpoS* and *gyrA*; twenty-three *Dickeya* strains were included in a phylogenetic analysis with a neighbor joining algorithm and bootstrapped at 1,000 replications. (B) Calculation of ANI and AP between pairs of genome sequences of *D*. *zeae* strains. Upper numbers indicate the values of ANI, and lower numbers indicate the values of AP. (C) Numbers of common or specific genes among *D*. *zeae* rice strains in an orthoMCL analysis. Upper numbers indicate the number of groups of genes, and lower numbers in parentheses indicate the number of genes.

Phylogenetic analysis of concatenated sequences of nine genes distinctly separated *D*. *zeae* strains from the other nine species (Fig 4A). Among the *D*. *zeae* strains, strains EC1, ZJU1202, DZ2Q, and EC2 from rice were grouped into a clade differentiated from other *D*. *zeae* strains. However, EC2 was placed at a different branch from the previously identified strains from rice (Fig 4A). This was also demonstrated by the phylogenetic analysis based on whole-genome sequences (S2 Fig). ANI analysis indicated that the ANI values between EC2 and previously identified strains from rice ranged from 97.24% to 97.27% and reached the suggested cutoff (96.00%) for species delineation [42], but they were lower than the values between any two previously identified strains from rice (Fig 4B); AP values between EC2 and previously identified strains from rice were also based on lower alignment coverages ranging from 84.26% to 84.71%. Alternatively, EC2 also showed a close relationship with the other two *D*. *zeae* strains, NCPPB 3531 and CSL_RW192 (Fig 4A and 4B).These suggested that EC2 experienced a genetic divergence from previously identified *D*. *zeae* strains from rice.

### Genome dissimilarities between EC2 and other strains isolated from rice

Synteny analysis between EC2 and other complete *D*. *zeae* genomes indicated that EC2 was highly co-linear with *D*. *zeae* genomes, except for a number of genomic dissimilarities like inversion and rearrangement events (S3 Fig). Comparing the genomes of four strains from rice including EC2, EC1, ZJU1202, and DZ2Q, orthoMCL cluster analysis revealed that 3,298 orthologous gene groups containing 13,432 genes were conserved in all of the four strains (Fig 4C). There were 365 groups and 391 genes specific to EC2, and 236 groups and 717 genes conserved within the previously identified strains from rice, but not in EC2. The specific genes in EC2 included those in a CPS biosynthesis cluster, T6SS gene clusters, a subtype I-F CRISPR cluster, and an NRPs- PKs cluster similar to a known viscosin cluster (S1 Table). Genes absent in EC2 included those in an EPS biosynthesis cluster, a zeamine cluster, and *hrp*-type T3SS, T4SS, and T5SS clusters (S2 Table). The zeamine cluster in EC1 encodes the phytotoxins which have a toxic effect against rice seed germination [22]. Accordingly, both culture crude and culture extract of EC1 significantly inhibited rice seed germination, but those of EC2 and MS1 did not (S4 Fig).

### Secretion systems

**Fig 5:**
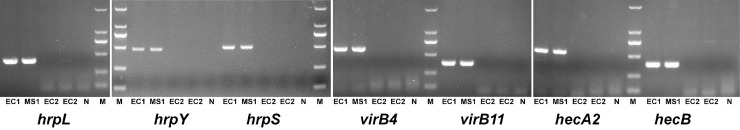
PCR detection of genes of T3SS, T4SS, and T5SS from *D*. *zeae* strains. *hrpL*, *hrpY*, and *hrpS* are genes of T3SS; *virB4* and *virB11* are genes of T4SS; *hecA2* and *hecB* are genes of T5SS. Lane M indicates molecular weight markers from top to bottom (2,000, 1,000, 750, 500, 250, 100 bp); lane EC1 indicates *D*. *zeae* EC1 from rice; lane MS1 indicates *D*. *zeae* MS1 from banana; lane EC2 indicates *D*. *zeae* EC2 from rice; and lane N indicates ddH_2_O template.

T1SS cluster composed of *prtG*–*prtA* (*DWV07_10200*–*DWV07_10235*) and *out-*type T2SS consisted of *outS*, *outB*–*outO* operon (*DWV07_13930*, *DWV07_13925*–*DWV07_13865*) were both conserved in EC2, but not *hrp*-type T3SS, T4SS, and T5SS. This was further confirmed by PCR, the genes of *hrpL*, *hrpY*, and *hrpS* of *hrp*-type T3SS, *virB4* and *virBll* of T4SS, and *hecA2* and *hecB* of T5SS of *D*. *zeae*, were not able to be amplified in EC2; we successfully amplified these gene products in EC1 and MS1 (Fig 5). This was consistent with orthoMCL cluster analysis. Compared with EC2, the previously identified strains from rice and other two close stains, NCPPB 3531 and CSL_RW192, had T3SS–T5SS, except that T4SS was not found in the genome of NCPPB 3531.

**Fig 6:**
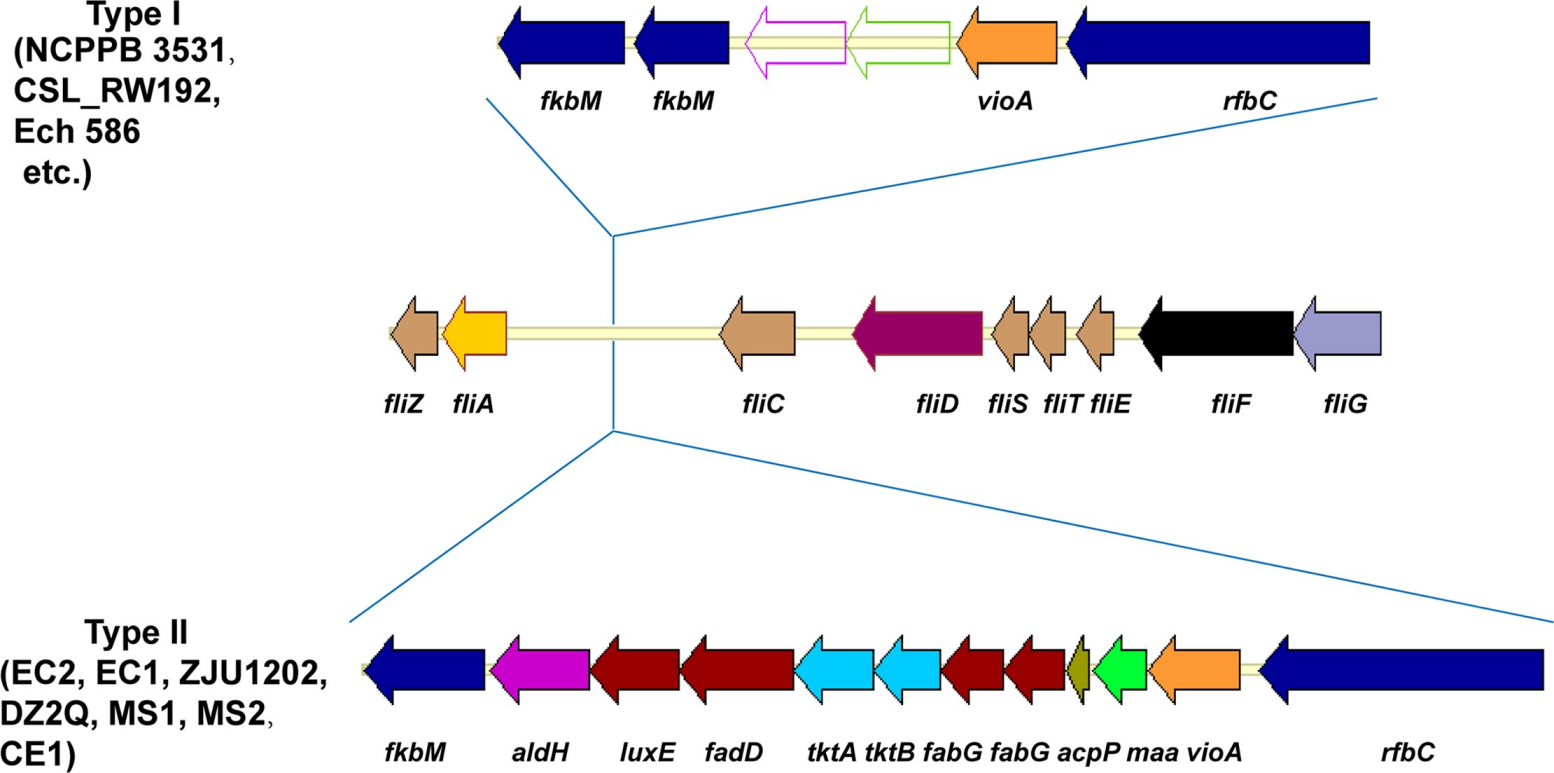
Two types of genomic organization found in the flagellar-type T3SS of *D*. *zeae* strains. Compared to other *D*. *zeae* srains, *D*. *zeae* strains from rice have a longer gene cluster between the genes of *fliA* and *fliC*, as do the other Guangdong strains from banana and *C*. *edulis*. Gene *fkbM* encodes a methyltransferase; *aldH*/*luxC*, *luxE*, *fadD*, f*abG*, *acpP* encode fatty acid biosynthesis proteins; *tktA* and *tktB* encode sugar biosynthetic proteins; *maa* encodes a maltose O-acetyltransferase; *vioA* encodes an aminotransferase; and *rfbC* encodes a glycosyltransferase.

The flagellar cluster considered a subtype of T3SS was found in EC2, including the flagellin gene *fliC*, 38 flagellar biosynthesis genes, 2 flagellar motor genes, and 7 chemotaxis-associated genes (EC2: *DWV07_12460*–*DWV07_12760*, spanning 60.6 Kb). This was conserved in EC1, ZJU1202, DZ2Q, NCPPB 3531, and CSL_RW192, plus other *D*. *zeae* strains like MS1 and Ech586 (Fig 6). Like EC2, all of the other Guangdong strains, including strains EC1 and ZJU1202 from rice, strains MS1 and MS2 from banana, and strain CE1 from *C*. *edulis*, harbored a gene cluster (*fkbM*-*aldH*-*luxE*-*fadD*-*tktA*-*tktB*-*fabG*-*fabG*-*acpP*-*maa*-*vioA-rfbC*) upstream of *flic*, as did the Italy strain DZ2Q isolated from rice (Fig 6). This gene cluster encoded glycosyltransferase RfbC; aminotransferase VioA; maltose O-acetyltransferase Maa; the sugar biosynthetic proteins TktA and TktB; the fatty acid biosynthesis proteins AcpP, FabG, FadD, LuxE, and AldH/LuxC; and methyltransferase FkbM. Unlike EC2, other strains in the genus *Dickeya* including NCPPB 3531, CSL_RW192, and Ech586, lacked maltose O-acetyltransferase, sugar biosynthetic proteins, or fatty acid biosynthesis proteins and contained only an *fkbM*-*fkbM*–*vioA*-*rfbC* gene cluster, where a carbamoyl-phosphate synthase gene and a dehydrogenase gene were inserted between *fkbM* and *vioA*. GC contents of the former type of larger gene clusters (37.02%–39.22%) were much lower than those of the latter types of shorter gene clusters (47.96%–48.30%).

**Fig 7:**
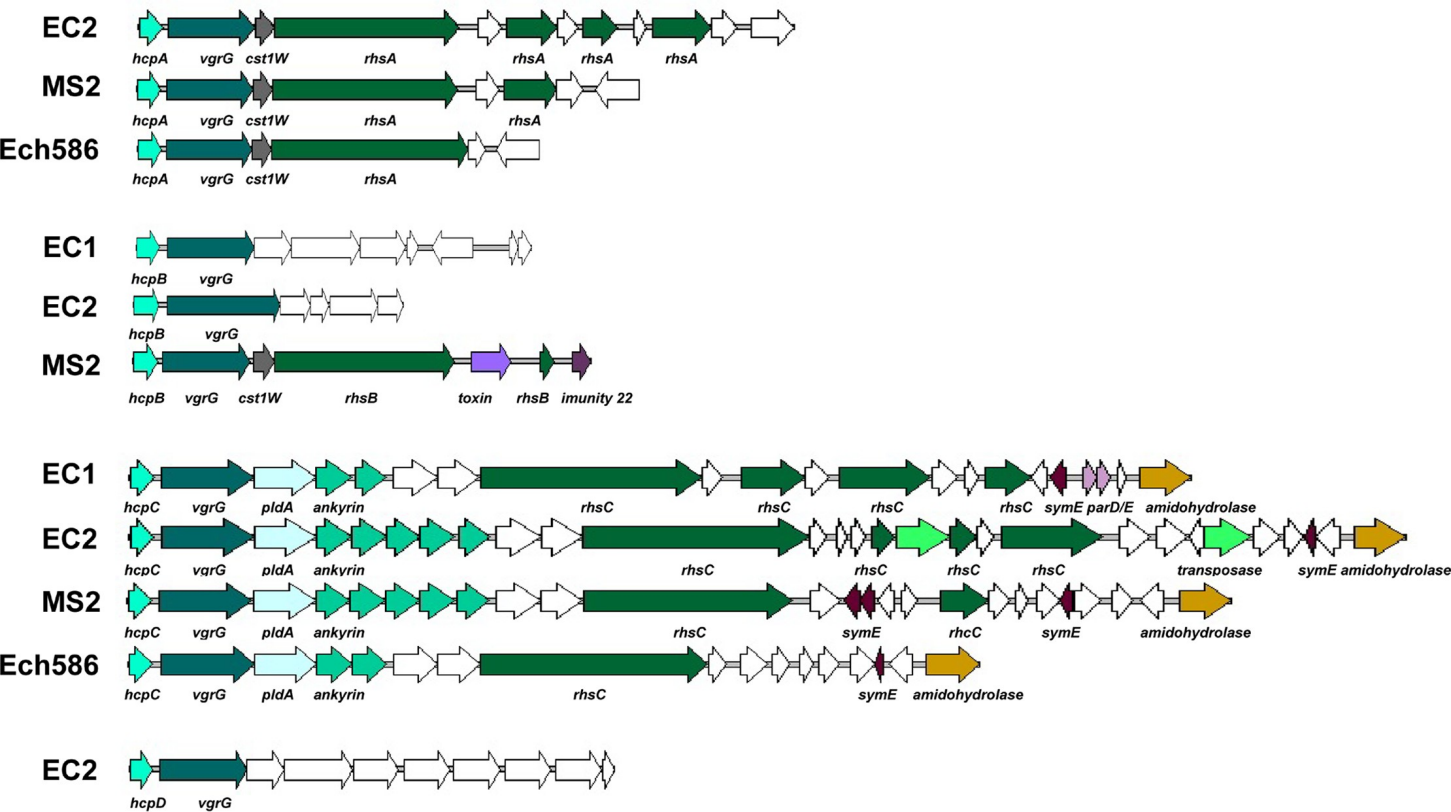
Genomic organization of the T6SS in *D*. *zeae* strains. The four *hcp* genes in *D*. *zeae* EC2 are at loci *DWV07_07765*, *DWV07_03820*, *DWV07_06350*, and *DWV07_18735*. Genes *hcp* and *vgrG* are present in pairs in T6SS and encode an extracellular substrate. The *rhs* genes encode nuclease effector proteins of T6SS. Homologous genes are presented in the same color.

A gene cluster encoding T6SS was also present in EC2 (*DWV07_06350*–*DWV07_06570*). The complete set of genes included: the hemolysin-coregulated gene (*hcp*) and valine-glycine repeat G gene (*vgrG*); rearrangement hotspot elements (*rhs*); and the core T6SS genes *impB*/*tssB*, *impC*/*tssC*, gp25 (lysozyme)/ tssE, *impG*/*tssF*, *impH/tssG*, filamentous hemagglutinin (FHA)/*tagH*, *vasD/tssJ*, *impJ*/*tssK*, *impK*, *clpB*/*tssH*, *sfa* (sigma-54-dependent Fis family), *vasI*/*tagO*, *impA /tssA*, *icmF*/*tssM*, *vasL*, and tetratricopeptide repeats (TPRs). This T6SS cluster was also found in the other strains from rice, EC1, ZJU1202, and DZ2Q, as well as other *D*. *zeae* strains like MS2 and Ech586 (Fig 7). Among these T6SS genes, *hcp* and *vgrG* not only existed within this gene cluster, but they also had homologous genes at other genomic positions. The number of pairs of *hcp* and *vgrG* varied among EC2 and other *D*. *zeae* strains. For example, EC2 had four, while MS2 had three and EC1 and Ech586 each contained two pairs. Compared to EC2 and MS2, EC1 did not have an *hcpA*-*vgrG* locus and Ech586 did not have an *hcpB*-*vgrG* locus; besides, the fourth *hcp*-*vgrG* locus (*hcpD*-*vgrG*) was found in the genome of EC2 rather than EC1 or MS2 or Ech586 (Fig 7). Furthermore, locus *hcpD*-*vgrG* was only found within EC2 among all of the available *D*. *zeae* genomes.

Among these *hcp*-*vgrG* loci, only *hcpC*-*vgrG* contained a complete set of T6SS core genes, and only some of them were linked to the *rhs* gene or gene cluster (Fig 7). As with EC1, EC2 did not contain the *rhs* gene or gene cluster at the locus of *hcpB*-*vgrG*, nor at *hcpD*-*vgrG*. EC2 contained the *rhs* genes at the loci of *hcpA*-*vgrG* and *hcpC*-*vgrG*. MS2 have the *rhs* genes at all of the loci of *hcpA*-*vgrG*–*hcpC*-*vgrG*. In another case, these strains varied in the number of additional orphan *rhs* toxin-immunity pairs downstream of the main *rhs* genes. EC2 harbored three more *rhs* genes in tandem arrays downstream of both *rhsA* and *rhsC*, EC1 also shown three additional *rhs* genes downstream of *rhsC*, and MS2 contained one additional *rhs* gene downstream of all three *rhs* loci, while Ech586 possessed only the main *rhs* genes at the loci of both *rhsA* and *rhsC* (Fig 7). However, the main *rhs* genes, *rhs*As, *rhsB*s, *rhsCs*, in the different *D*. *zeae* strains significantly differed in their C-terminal toxin domains (Rhs-CTs). These genes contained extensive polymorphisms at the C-terminal toxin moiety and encoded conserved large N-domain YD-peptide repeats.

### EPS and CPS biosynthesis clusters

**Fig 8:**
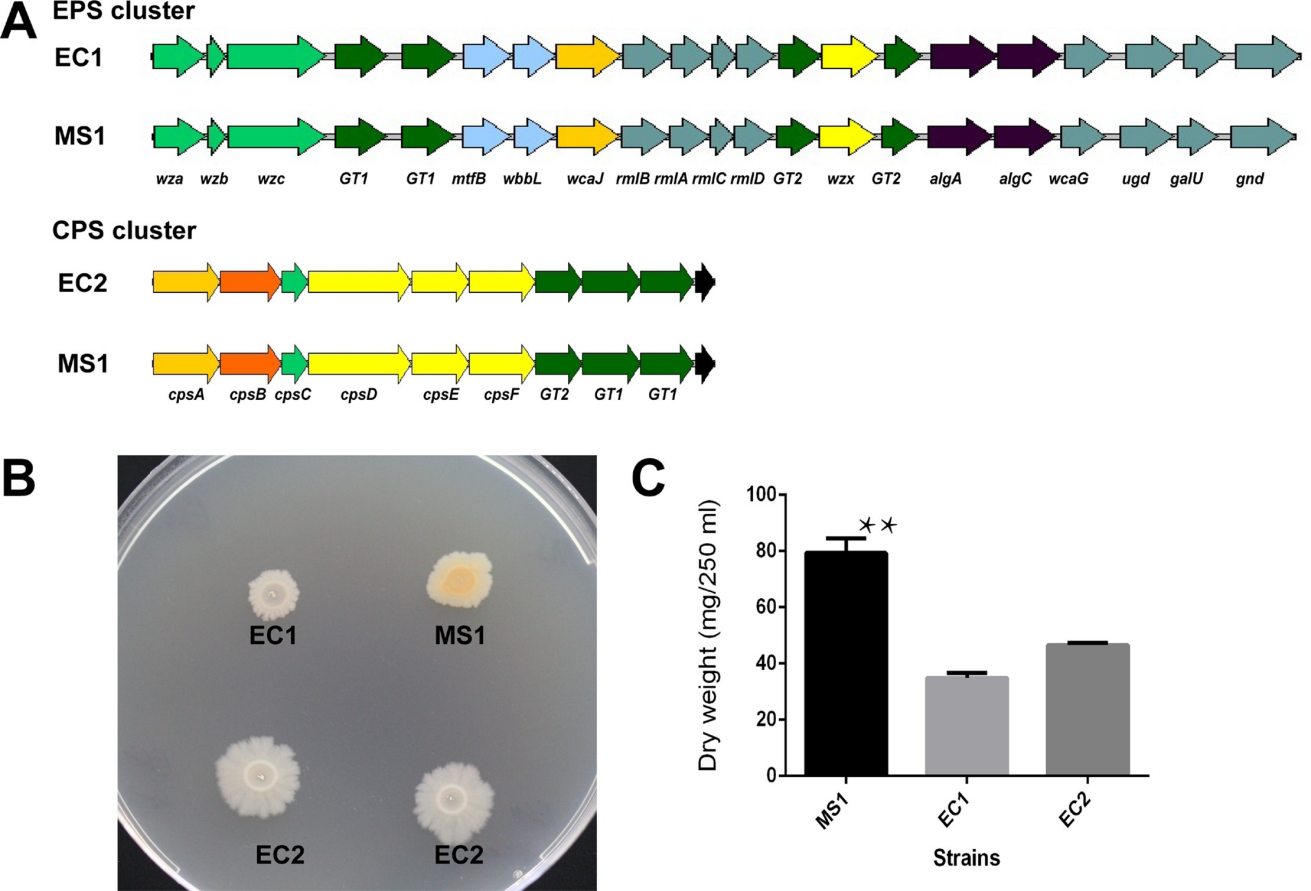
EPS and CPS biosynthesis clusters in *D*. *zeae* strains and their EPS production. (A) Genomic organization of EPS and CPS biosynthesis clusters in *D*. *zeae* strains EC2, EC1, and MS1. Strain from banana, MS1, has both EPS and CPS clusters. Previously identified strains from rice, EC1, ZJU1202, and DZ2Q, only have the EPS cluster, and the newly identified strain from rice, EC2, only has the CPS cluster. Homologous genes are presented in the same color. (B) Colony morphology of *D*. *zeae* strains, EC2, EC1, and MS1, on NA plates supplemented with 2% glucose. Colonies of strain from banana, MS1, were more prominent than the flat colonies of strains from rice, EC1 and EC2. Colonies of EC1 grew more slowly and EC2 had obvious serrated borders. (C) Dry weight of EPS produced by *D*. *zeae* strains EC2, EC1, and MS1. ** indicates significant difference observed between MS1 and either of strains from rice, EC1 and EC2, by two-tailed t-test, and the p values of difference statistics were 0.0002 and 0.0004, respectively.

An EPS and CPS biosynthesis clusters were found in the genomes of *D*. *zeae* strains, but strains from rice did not have both of them. Taking *D*. *zeae* MS1 as an example, the EPS cluster in MS1 consisted of a large operon of 21 genes (*J417_RS0113995 –J417_RS0114100*), while the operon of the CPS cluster had 10 genes (*J417_RS0103105*–*J417_RS010315*0) (Fig 8A). Three *D*. *zeae* strains from rice, EC1, ZJU1202, and DZ2Q, lacked the CPS cluster but contained the EPS cluster (EC1: *W909_RS06075*– *W909_RS06175*; ZJU1202: *WYU_RS0108445*–*WYU_RS0108340*; DZ2Q: *J134_RS12880*–*J134_RS12780*) (Fig 8A), as did *D*. *zeae* CSL RW192. In a different instance, the new strain from rice, EC2, was the only *D*. *zeae* strain that lacked the EPS cluster, but harbored the CPS cluster (*DWV07_02695*–*DWV07_02740*). EPS cluster in *D*. *zeae* strains included genes encoding the polysaccharide export protein Wza, glycosyl transferase family proteins GT1 and GT2, undecaprenyl-phosphate glucose phosphotransferase WcaJ, polysaccharide biosynthesis protein Wzx, and others. Within CPS cluster, the predicted functions of some genes were similar to the EPS genes, such as *cpsA* encoding undecaprenyl-phosphate glucose phosphotransferase like *wcaJ*, *cpsC* encoding polysaccharide export protein like *wza*, *cpsE* encoding multidrug and toxic compound extrusion family and similar proteins in polysaccharide biosynthesis like *wzx*, as well as genes encoding glycosyl transferase family proteins GT1 and GT2. Further, the CPS cluster encoded another two capsular polysaccharide biosynthesis proteins, CpsD and CpsF, and an OM_channel superfamily protein CpsB. On the 2% glucose-supplemented NA plates, MS1 showed different colony morphology in comparison to EC1 and EC2. The colonies of MS1 were more prominent than the other two strains. EC1 showed the smallest colonies and EC2 exhibited colonies with a distinct, serrated border, indicating different EPS-associated phenotypes (Fig 8B). In turn, significantly more EPS was produced by MS1 than by those two strains from rice (Fig 8C).

## Discussion

### *D*. *zeae* strain EC2 was closest to previously identified strains from rice, but did not belong to same subgroup

Among the strains from rice, the previously identified strains from rice in China and Italy were much more conserved, except for EC2. Pairwise isDDH analysis (S5 Fig) found that these previously identified strains from rice reached the cut-off (90.00%) for a subspecies [43], and they were also suggested to be a subspecies within *D*. *zeae* [15].On other hand, ANI and AP analyses suggested EC2 was also close to the other two strains from non-rice hosts, NCPPB 3531 and CSL_RW192 (S5 Fig). In accordance with this, strains EC1, ZJU1202, DZ2Q, NCPPB 3531, and CSL RW192, were classified into a genomospecies named *D*. *oryzae* [44]. However, most of the genome sequences used in ANI and AP analyses were draft genomes, and ANI and AP analyses were based on pairwise comparisons. Thus, the comprehensive phylogenetic analyses using concatenated sequences of multiple genes of multiple stains might be better for explaining their genetic relationships and classification. Moreover, ANI and AP analyses showed EC2 and other strains from rice had higher similarities than strains from non-rice hosts. Thus, EC2 was a distant strain from the conserved subgroup of previous identified strains from rice, but still a member of the group of strains isolated from rice.

### Fatty acid and sugar clusters inserted within *fli*/*che* locus might be associated with flagellin glycosylation and its modification

Flagellin FliC contained a motif similar to Flg22 that is implicated in plant recognition. Similar to DspE, this flagellin protein is considered a cause of plant cell death in *Pectobacterium* spp. [45]. Further, flagellin glycosylation is ubiquitous in most phytopathogenic bacteria, including *D*. *dadantii* strains, and is important for the virulence of some phytopathogens [46]. Glycosyltransferase plays key roles in the formation of glycosylation protein by catalyzing the transfer of a saccharide onto the acceptors [47] and is used in the glycosylation of flagellin [48]. For *D*. *dadantii* 3937, mutation of glycosyltransferase RfbC (*Dda3937_03424*) affected the glycosylation of Flic and further diminished its pathogenicity on chicory plants. The genes *rfbC*–*fkbM* (located between *fliA* and *fliC*) were supposedly involved in flagellin glycosylation [49].

Among the Guangdong strains (strains from rice, EC1, EC2, and ZJU1202, strain from banana, MS1, and strain from *C*. *edulis*, CE1), and the Italy strain DZ2Q from rice, their *rfbC*–*fkbM* clusters were inserted with genes encoding maltose O-acetyltransferase, sugar biosynthetic proteins, and fatty acid biosynthesis proteins. This is consistent in that the glycosyltransferase and sugar biosynthetic genes responsible for flagellin glycosylation are usually adjacent to the flagellin gene [50]. Moreover, the flagellin glycan chains of some bacteria can be methyl-, formyl-, acetyl-, or amino-modified [51]. Their essential role in flagellin glycosylation, the modification of flagellin glycan, is important for other virulence factors like motility, exopolysaccharide production, and biofilm formation [52]. Compared to the previously explored *D*. *dadantii* gene cluster, the additional sugar biosynthetic and fatty acid biosynthesis genes might play essential roles in flagellin glycosylation. Of note, several fatty acid genes were involved in this cluster, suggesting that the flagellin glycan of these *D*. *zeae* strains may incorporate the liposugar component [51]. The versatility and complexity of the flagellin glycan structure in these pathogens might help them adapt to monocotyledonous hosts like rice, banana, and *C*. *edulis*. Interestingly, these strains from rice, banana, and *Canna* were mostly from Guangdong province, China, except for DZ2Q from Italy. Using the sequences of this inserted gene cluster among strains from rice, banana, and *Canna*, phylogenetic analysis revealed that EC2 was closer to other strains from rice. Those four strains from rice had 320 polymorphic sites and 0.020 of nucleotide diversity (*Pi*), whereas the other three strains from non-rice hosts showed 1,010 polymorphic sites and 0.085 of nucleotide diversity (*Pi*). The strains from non-rice had much higher genetic diversity, suggesting they were older than the strains from rice. Additionally, the observation of lower GC contents indicating the inserted gene cluster might be acquired by horizontal gene transfer (HGT), and these strains with this inserted gene cluster might differentiate from a same evolutionary event and were closely related. Thus, the *D*. *zeae* strains from rice might differentiate from *D*. *zeae* strains infecting other hosts in adjacent geographical areas, and retained this genomic characteristic either in the previously identified strains from rice in China and Italy or the newly identified strain from rice. This also indicated that the strains from rice was related to those other *D*. *zeae* strains carrying the similar inserted gene cluster and it might not be appropriate to simply classify the strains from rice into the genomospecies *D*. *oryzae* and to keep them apart in this study.

### T6SS was a potential important virulent factor of the newly identified strain from rice

T3SS–T5SS were not found in the newly identified strain from rice, EC2. These three secretion systems could be vital for the virulence of soft-rot *Pectobacteriaceae*, including *Dicke*ya pathogens [53–55]. *Pectobacterium* strains naturally lacking T3SS have been isolated, suggesting that T3SS is not required for their survival [56, 57]. This is also common in *D*. *paradisiaca* strains. The functions of T3SS in *Dickeya* strains are different from the “stealth” mechanisms of T3SS used by *Pseudomonas syringae* or other plant-pathogenic bacteria to attack plants [58]. However, the method that soft-rot strains in the *Pectobacteriaceae* use to compensate for these lost secretion systems in the plant infection process remains unknown [45].

T1SS, T2SS, and T6SS were conserved in all analyzed *D*. *zeae* genomes. T1SS-secreting metalloproteases can affect plant cell wall proteins or the activities of pathogen-secreting enzymes [59]. T2SS-secreting plant cell wall-degrading enzymes (PCWDEs) are a group of enzymes active in plant cell-wall digestion [60]. Mutants of T1SS showed delayed symptom expression [59] and mutations in *out*-type T2SS inhibited soft rot symptoms [61], indicating important roles of these two secretion systems in pathogenesis. Moreover, T2SS was conserved among different *Dickeya* species, including *D*. *paradisiaca*, as PCWDEs were considered “brute force” in creating soft rot symptoms [62].

Genetic elements besides T1SS and T2SS, however, are required for the efficient colonization of host plants and adaption to new growth conditions encountered in the hosts [58]. T6SS is the most recently discovered secretion system in gram-negative bacteria [63] and is well conserved among them [64]. T6SS has been shown to mediate inter-bacterial antagonism through competition with other bacteria for space and resources [65] and facilitates pathogen invasion through the killing of commensal species [66], and could also affect the production of EPS and reduce virulence [67]. For *Dickeya* pathogens, there are very few reports on the functional analyses of T6SS components. Rhs effector proteins in *D*. *dadantii* 3937 carry nuclease domains that degrade target cell DNA and play roles in contact-dependent growth inhibition [68], as is consistent with the important function of T6SS in inter-bacterial antagonism [65]. In this study, a diverse repertoire was observed in the T6SS Hcp-VgrG component and Rhs effectors among *D*. *zeae* strains. The Hcp-VgrG component is an essential part of T6SS, which is the extracellular substrate activating T6SS [69]; it is also always present in the same operon with Rhs effectors and interacts with those effector proteins [65]. For example, VgrG is required for RhsB-mediated inhibition in *D*. *dadantii* 3937 [68]. Thus, those differentiations within T6SS loci might contribute to their virulence by enabling them to compete more effectively with other host-associated bacteria and exploit a specific host niche [65, 70].

The term effective competition applies especially to the newly identified strain from rice, EC2, which did not have the zeamine or T3SS clusters. Besides the inter-bacterial antagonism of T6SS, T6SS is closely related to T3SS in bacterial pathogens. The transition between T3SS and T6SS could coordinate bacterial lifecycles, like planktonic and biofilm formation in *Pseudomonas aeruginosa*, and production of these secretion systems could be switched by RetS/GacS sensors and the second messenger c-di-GMP [71]. Moreover, mutation of the T6SS core gene, *tssM*, also affects the expression of T3SS genes in *Ralstonia solanacearum* [72]. Collectively, T6SS might play important roles in the pathogenesis of EC2, such as effective competition with host-associated bacteria and adaption to the host environment. Its mechanism of pathogenesis might be different than in previously isolated strains from rice and require further exploration in the future. EC2 could be a potential type strain for the investigation of T6SS functions independent of the effects of other virulence determinants, like T3SS.

### Loss of EPS or CPS biosynthesis cluster might be associated with EPS production and biofilm phenotypes

EPS is a major virulence factor, increasing the disease severity of bacterial pathogens in their hosts [73]. In this study, EPS cluster found in previously identified strains from rice was homologous with the EPS biosynthesis cluster (*wza*-*wzb*-*wzc*-*wzx*) encoding the group 1 and 4 capsules in *Escherichia coli* [74]. This locus was also considered in relation to the EPS biosynthesis of *Erwinia pyrifoliae* [75] and *Pectobacterium brasiliense* [76]. EPSs have two types of polysaccharides: one released as an exopolysaccharide (EPS), or slime polysaccharide, and the other as a capsular polysaccharide (CPS) that can form a discrete capsule around the cell and is intimately associated with the cell surface [77]. A CPS cluster was found in EC2 rather than EC1 and other strains from rice. Among gram-negative bacteria, EPS and CPS are often essential virulence determinants in plant pathogens, and the processes of their biosynthesis and export are indistinguishable [77], some of CPS genes were also predicted to play the functions similar with EPS genes in this study. Thus, loss of one of them might cause EC1 and EC2 to produce significantly less EPS than MS1 (Fig 8), as was consistent with the Hu’s observation that appreciably less EPS was produced by EC1 than other *D*. *zeae* strains [16].

EPS is a major component of the bacterial biofilm matrix, forming the scaffold for the biofilm architecture. It is also responsible for adhesion to surfaces and the cohesiveness of biofilm [78]. In this study, we found that only MS1 could form intact, regular biofilm on biofilm-inducing SOBG medium. EC1 biofilm appeared clumps, and EC2 biofilm was thinner (S6 Fig). These results indicated that the loss of either the EPS or CPS cluster could limit biofilm formation by affecting the EPS-associated phenotype. The effect on biofilm formation was further demonstrated by the different colony morphology on Congo red (CR; indicative of curli fibers and cellulose production) plates or Calcofluor (CF; indicative of cellulose production) plates. Curli fibers and cellulose synthesis have been considered a primary cause of biofilm formation [79]. We found that MS1 and EC2 formed wrinkled, red colonies on CR plates while EC1 produced smooth blue colonies (S6 Fig). These three strains all fluoresced on CF plates under UV conditions (S6 Fig). Thus, we speculated that loss of the CPS cluster in EC1 might cause defective curli production, limiting the functional amyloids that form the extracellular matrix of biofilms [80].

### An evolutionary pathway for the new strain EC2 isolated from rice was different from previously identified strains

CRISPRs protect against bacteriophages and plasmids, and are widely distributed among bacteria [81]. Two types were found in most *D*. *zeae* strains. CRISPR-1 (subtype I-F) consisted of CRISPR-associated (Cas) core proteins (Cas1, Cas3) and Csy proteins (Csy1–Csy4), and CRISPR-2 (subtype I-E) contained the Cas core proteins (Cas1, Cas2, Cas3, Cas5) and Cse proteins (Cse1–Cse4). In a previous study, we found the CRISPR-1 array was conserved in *D*. *zeae* species, but the strains from rice contained either one simple direct repeat sequence or lacked the whole array [82]. Strain EC2, however, made this differentiation more complicated as it had the complete CRISPR-1 array (position: 3,690,316–3,709,212 bp) at the allele locus.

Six PKs/NRPs gene clusters were predicted in EC1, including gene clusters related to the biosynthesis of zeamine, indigoidine, bicornutin, siderophore, O-antigen saccharide, and turnerbactin. These PKs/NRPs clusters were also found in EC2, except for the zeamine cluster (*zmsO*–*zmsN*). It was only found in the *D*. *zeae* strains from rice, ZJU1202 and DZ2Q, *D*. *solani* strains, and *D*. *fangzhongdai* strains [15, 82]. The non-acquisition of the zeamine cluster encoding the phytotoxins which affected the rice seed germination, however, did not affect the pathogenicity of EC2 on rice plants, indicating the zeamine cluster might not be a necessary virulence factor for this new strain from rice.

CRISPR-1 is universal in all *Dickeya* species and might have been acquired before the differentiation of the genus *Dickeya* [82]. Lower genomic GC contents revealed that the zeamine clusters were likely derived from HGT [15]. Thus, strains from rice in China and Italy, EC1, ZJU1202, and DZ2Q, were much conserved, and all of them lost CRISPR-1 and acquired the zeamine cluster late in their evolutional process. In another case, missing of the T3SS–T5SS and EPS cluster, retention of the CRISPR-1 array, and failure of the zeamine cluster in EC2 suggests that EC2 was likely on an evolutionary pathway different to previously identified strains infecting rice. However, EC2 was still phylogenetically closest to that subgroup of strains previously isolated from rice. This was also indicated by the closer relationship between EC2 and other strains from rice in the genomic trait of *fli*/*che* loci incorporated with potential flagellin glycosylation and modification, in comparison with the other strains from non-rice hosts.

## Conclusions

We isolated a new strain of *D*. *zeae*, EC2, from rice plant with typical rice foot rot symptoms. It was different from previously identified causal agents in biochemical characteristics and seed germination inhibition capability. Compared to the genomes of other strains from rice, this new strain lacked the *hrp*-type T3SS, T4SS, T5SS, and zeamine clusters, contained only a CPS cluster rather than the customary EPS cluster, and retained a subtype I-F CRISPR cluster. These differences indicated EC2 was likely on an evolutionary pathway different to the conserved subgroup consisted of previously identified strains from rice in China and Italy. However, EC2 was still phylogenetically closest to other *Dickeya* strains from rice, indicating all strains from rice have evolved toward a same direction for the adaption to rice plants. They retained common virulence traits on rice, like the characteristic *fli*/*che* loci and the varied T6SSs. Thus, we report a new type of foot rot pathogen in the rice fields of China that requires our future attention.

## Supporting information

S1 Fig Principal co-ordinates analysis of the biochemical characteristics among *D*. *zeae* strains. The analyzed strains include strains from rice, EC1 and EC2, strain from banana, MS1, and strain from *C*. *edulis*, CE1. Biochemical characteristics were based on their use of different substrates and obtained from Biolog analysis.
(TIF)

S2 Fig Phylogenetic analysis of *D*. *zea*e strains based on complete or draft genome sequences. *Dickeya dadantii* 3937 was used as the out-group control.
(TIF)

S3 Fig Synteny analyses between EC2 and other *D*. *zea*e strains with complete genome sequences. MS2 is a strain from banana in Guangdong, China. Ech586 is a strain from *Philodendron* Schott in Florida, USA. Red indicates the homologous regions present in the same orientation; blue indicates the homologous regions present in an inverted orientation.
(TIF)

S4 Fig Inhibitory activity on rice seed germination by *D*. *zeae* strains. The analyzed strains include strains from rice, *D*. *zea*e EC1 and EC2, strain from banana, *D*. *zea*e MS1. (A) Inhibitory activity of culture crude from different strains. (B) Inhibitory activity of culture extract from different strains. CK indicates negative control.
(TIF)

S5 Fig Calculation of isDDH between pairs of genome sequences of *D*. *zeae* strains. The single genome-to-genome distance value was calculated at the GGDC web service (http://ggdc.dsmz.de/ggdc.php) using formula 2.
(TIF)

S6 Fig Characteristics associated with biofilm formation of *D*. *zeae* strains. (A) Phenotypes of CR-binding strains from rice, EC1 and EC2, and strain from banana, MS1. (B) Phenotypes of CF-binding strains from rice, EC1 and EC2, and strain from banana, MS1. CR and CF plates were incubated at 25°C for 4 d. (C) Biofilm formation after growth in SOBG medium for 24 h. (D) Biofilm formation after growth in SOBG medium for 48 h. The static SOBG cultures were incubated at 30°C for 24 to 48 h.
(TIF)

S7 Fig (PDF)

S1 Table Specific genes of *D*. *zeae* EC2 in comparison with previously identified strains from rice. The previously identified strains from rice include *D*. *zeae* EC1, ZJU1202, and DZ2Q. The 391 specific genes were grouped into 365 orthoMCL groups.
(XLSX)

S2 Table Specific genes of previously identified strains from rice in comparison with *D*. *zeae* EC2. The previously identified strains from rice include *D*. *zeae* EC1, ZJU1202, and DZ2Q. The 717 specific genes were grouped into 236 orthoMCL groups.
(XLSX)
