# Supplementary figures and images for: Genomic divergence between Dickeya zeae strain EC2 isolated from rice and previously identified strains, suggests a different rice foot rot strain
Source: PLoS One. 2020 Oct 20;15(10):e0240908. doi: 10.1371/journal.pone.0240908 (PMC7575072; doi:10.1371/journal.pone.0240908)

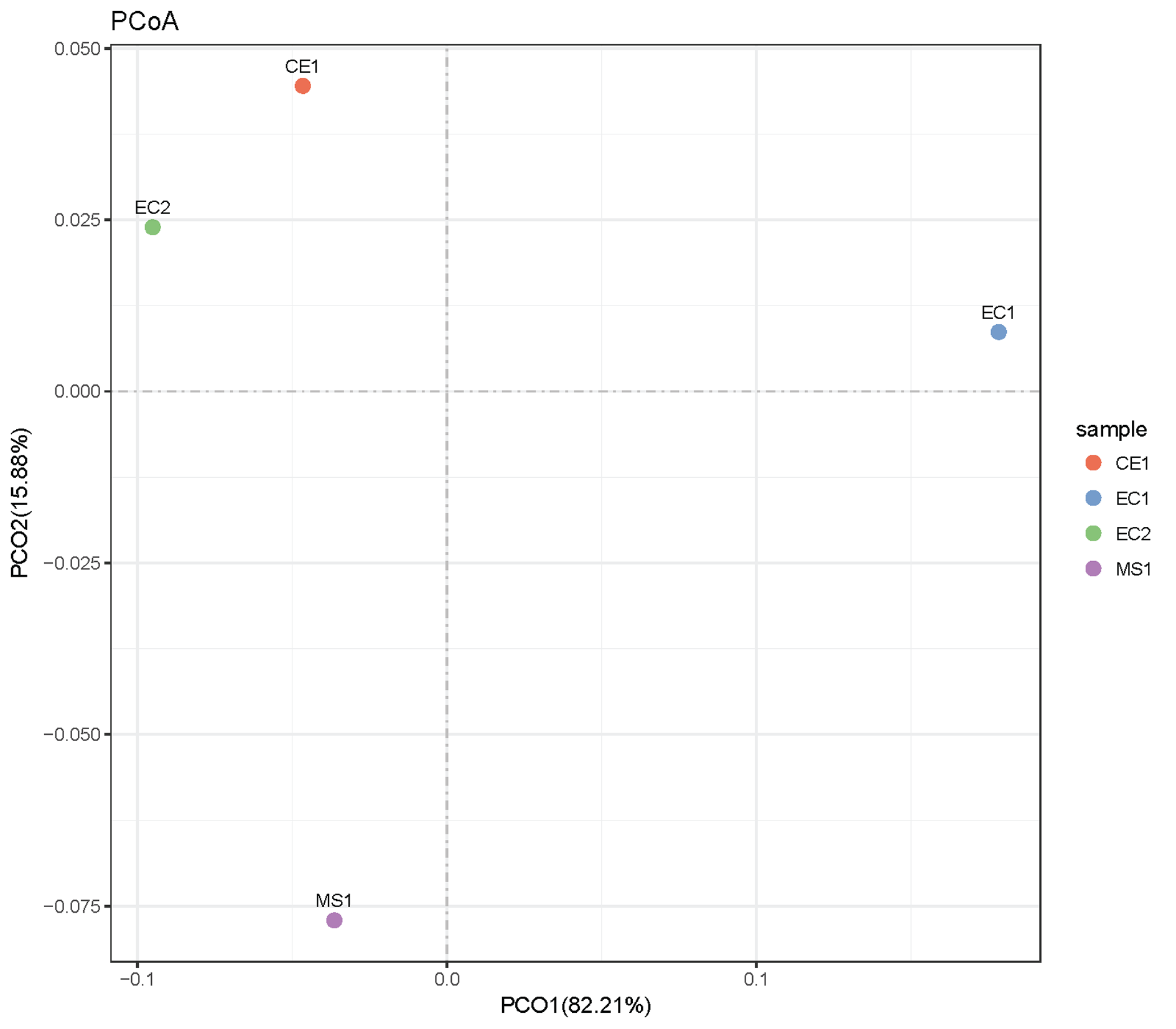

Supplement: S1 Fig — The analyzed strains include strains from rice, EC1 and EC2, strain from banana, MS1, and strain from C. edulis, CE1. Biochemical characteristics were based on their use of different substrates and obtained from Biolog analysis. (TIF) [file pone.0240908.s001.tif]

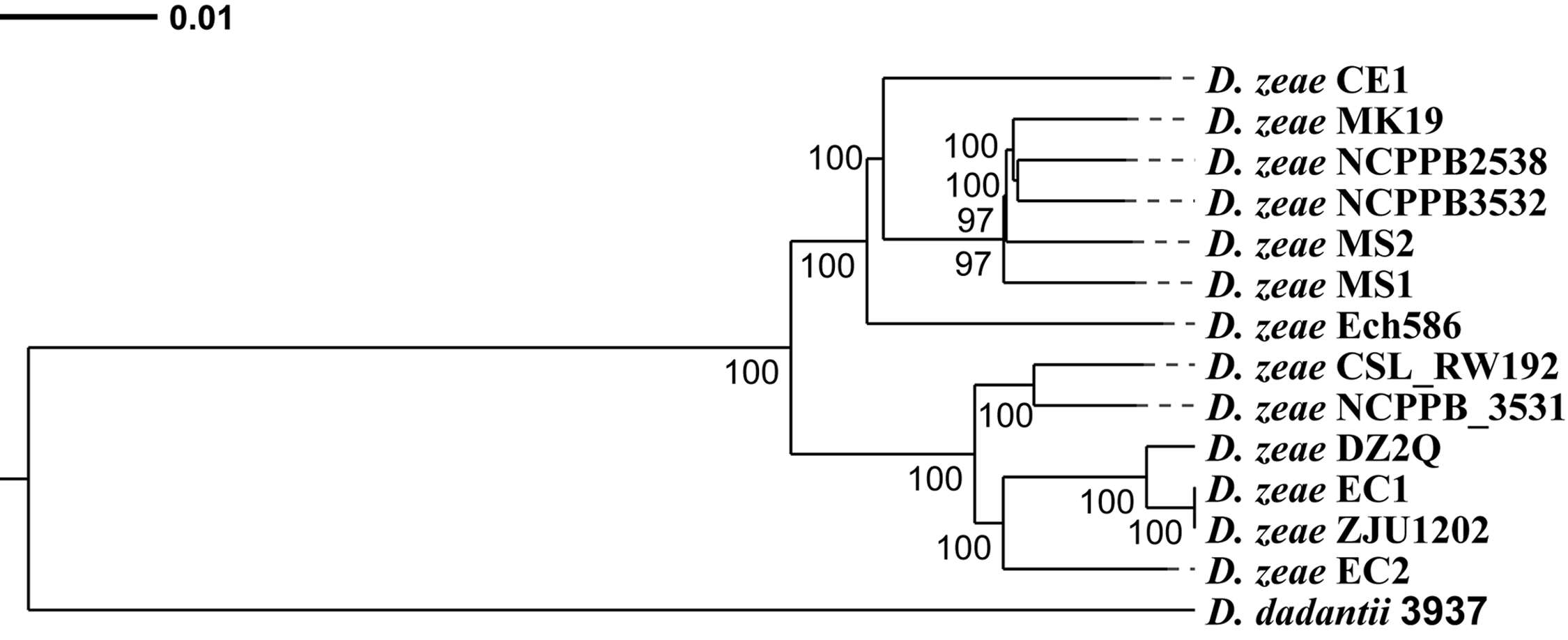

Supplement: S2 Fig — Dickeya dadantii 3937 was used as the out-group control. (TIF) [file pone.0240908.s002.tif]

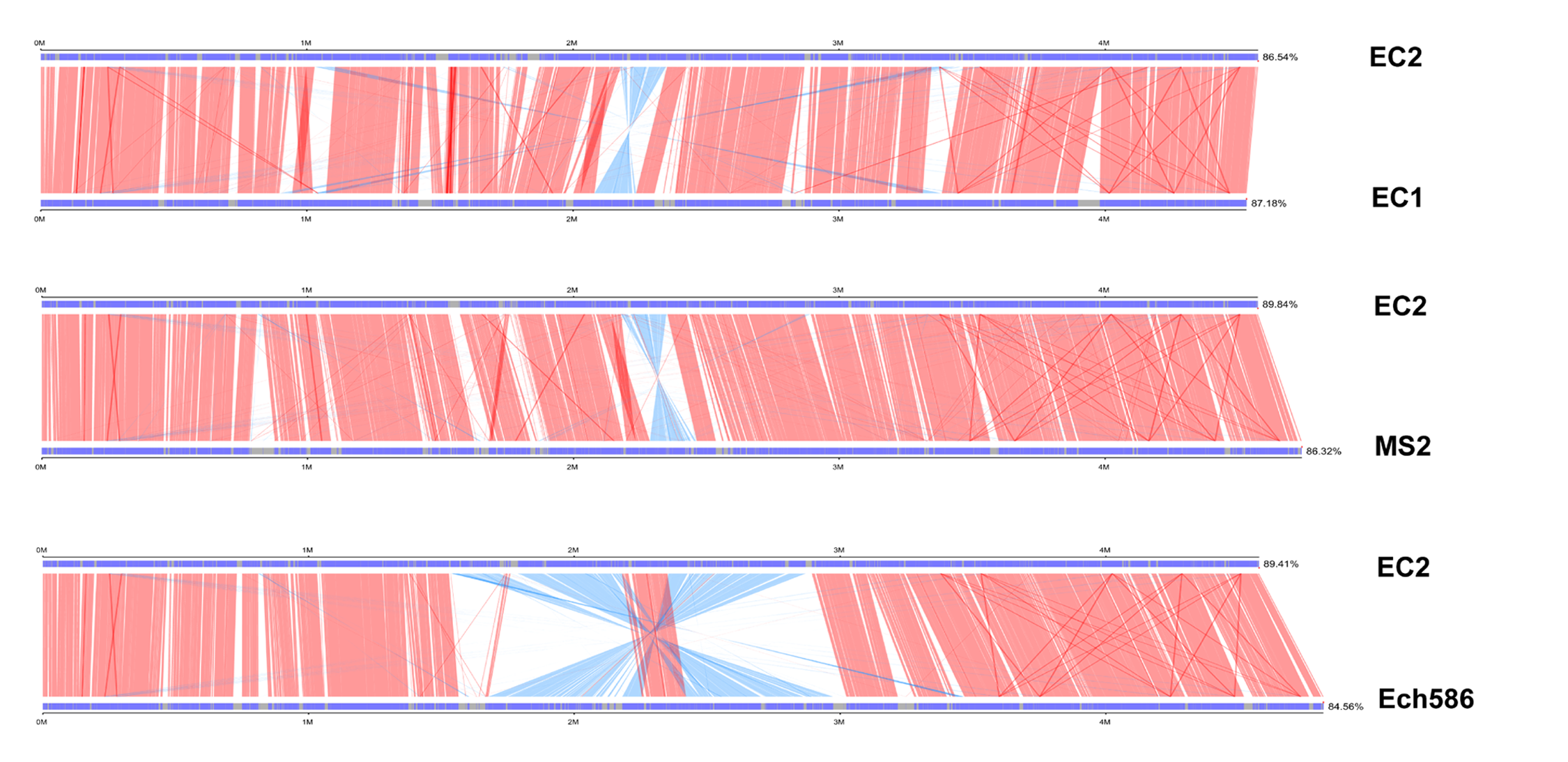

Supplement: S3 Fig — MS2 is a strain from banana in Guangdong, China. Ech586 is a strain from Philodendron Schott in Florida, USA. Red indicates the homologous regions present in the same orientation; blue indicates the homologous regions present in an inverted orientation. (TIF) [file pone.0240908.s003.tif]

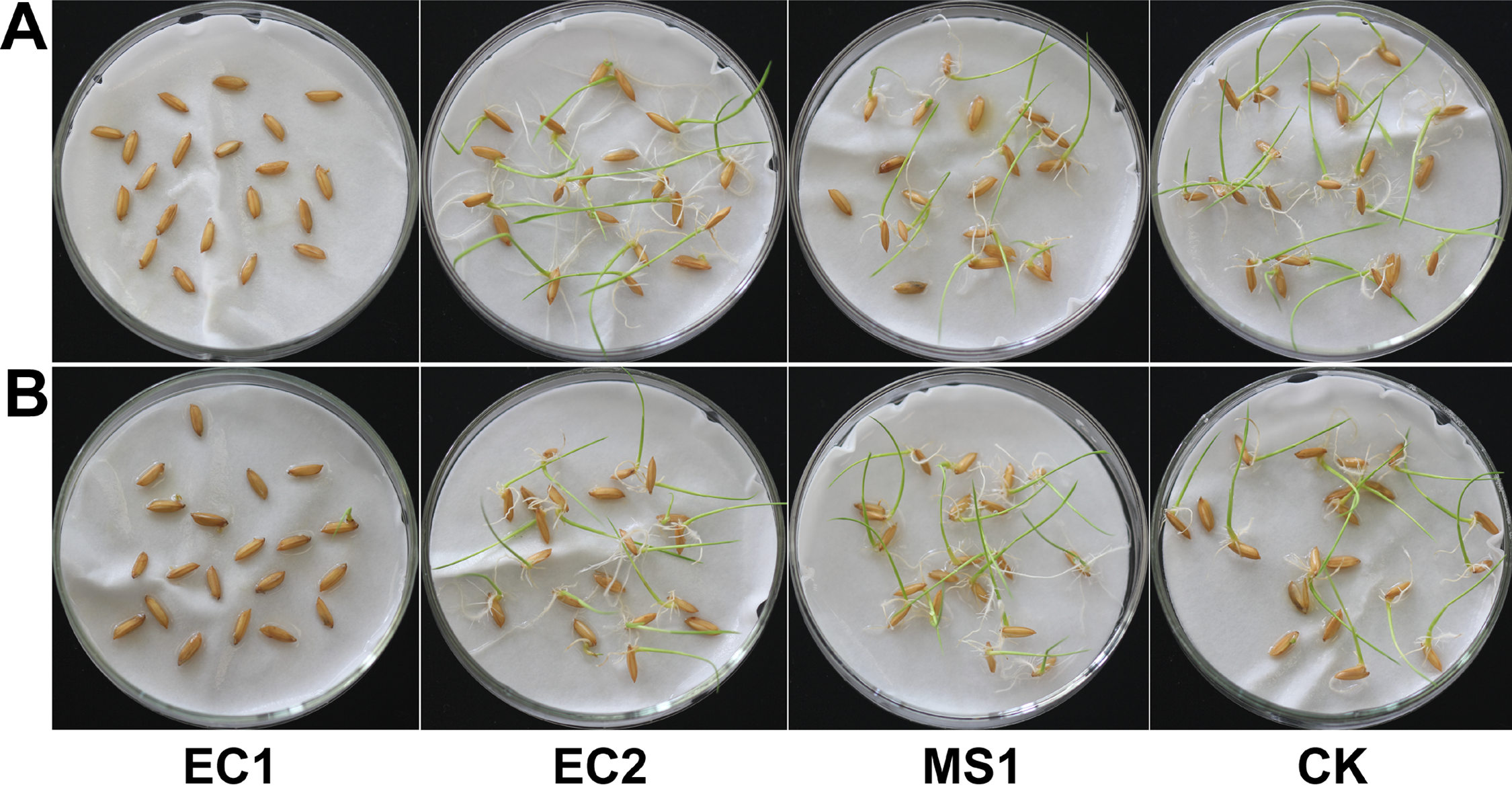

Supplement: S4 Fig — The analyzed strains include strains from rice, D. zeae EC1 and EC2, strain from banana, D. zeae MS1. (A) Inhibitory activity of culture crude from different strains. (B) Inhibitory activity of culture extract from different strains. CK indicates negative control. (TIF) [file pone.0240908.s004.tif]

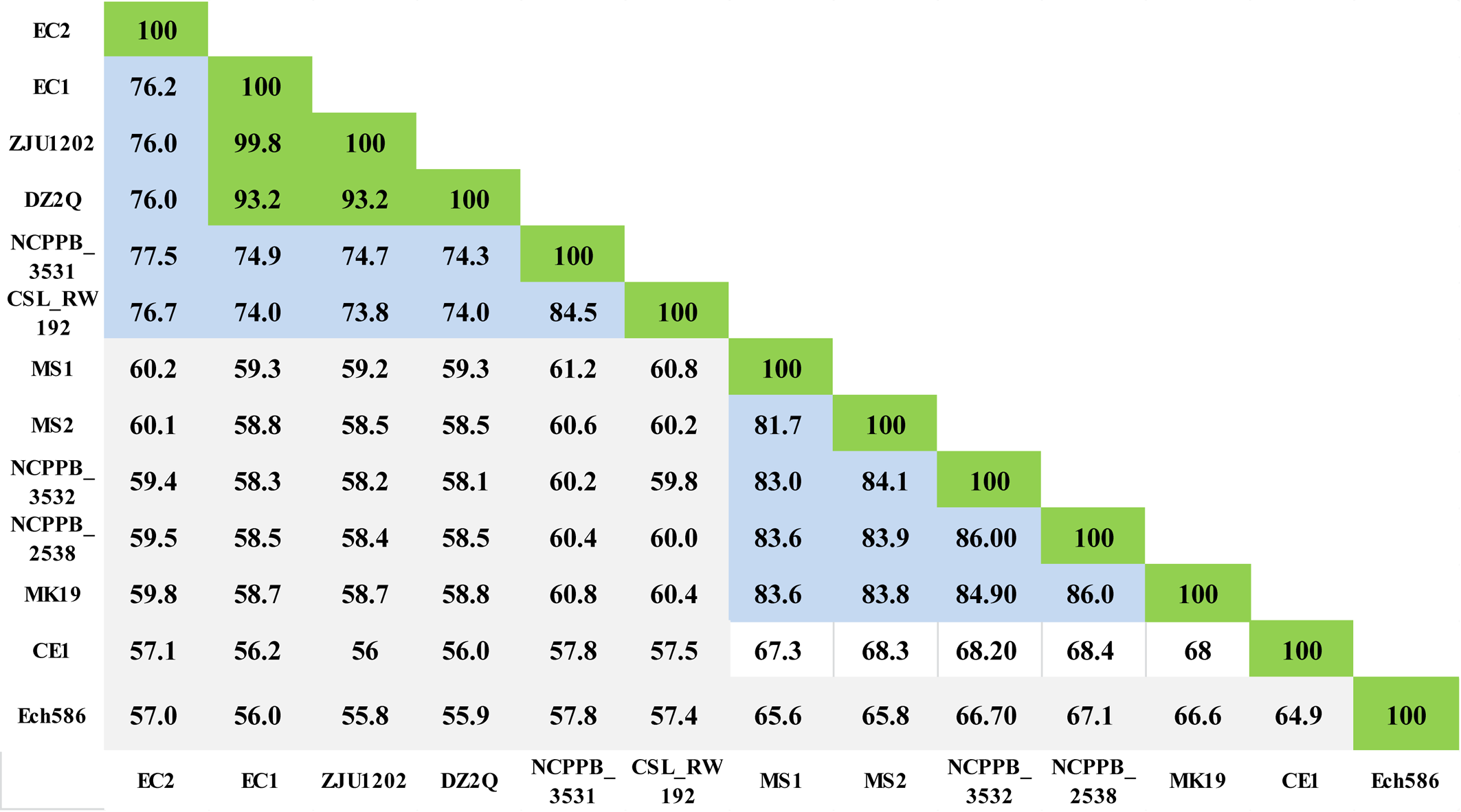

Supplement: S5 Fig — The single genome-to-genome distance value was calculated at the GGDC web service (http://ggdc.dsmz.de/ggdc.php) using formula 2. (TIF) [file pone.0240908.s005.tif]

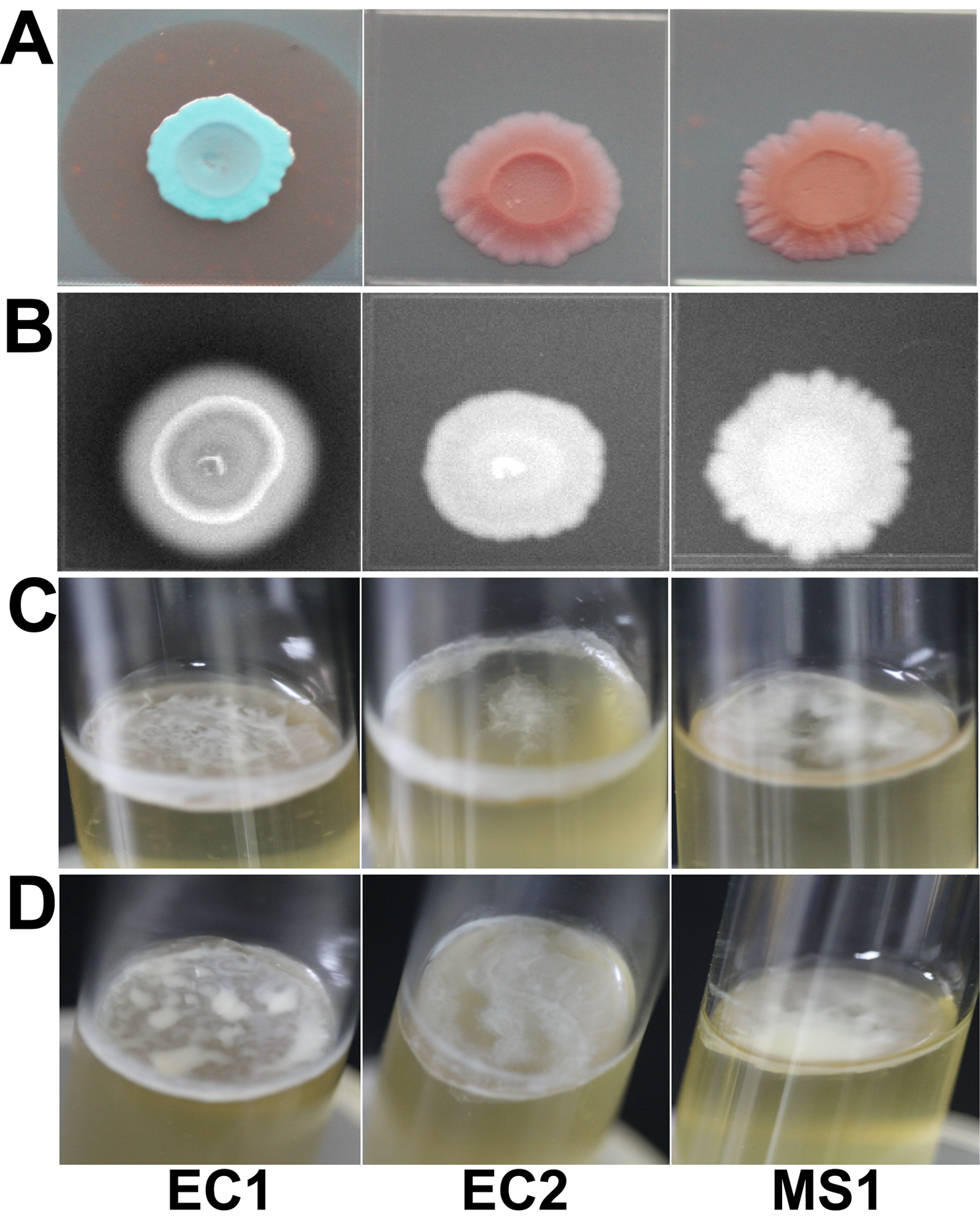

Supplement: S6 Fig — (A) Phenotypes of CR-binding strains from rice, EC1 and EC2, and strain from banana, MS1. (B) Phenotypes of CF-binding strains from rice, EC1 and EC2, and strain from banana, MS1. CR and CF plates were incubated at 25°C for 4 d. (C) Biofilm formation after growth in SOBG medium for 24 h. (D) Biofilm formation after growth in SOBG medium for 48 h. The static SOBG cultures were incubated at 30°C for 24 to 48 h. (TIF) [file pone.0240908.s006.tif]
