# Supplementary material for: Genomic divergence between Dickeya zeae strain EC2 isolated from rice and previously identified strains, suggests a different rice foot rot strain
Source: PLoS One. 2020 Oct 20;15(10):e0240908. doi: 10.1371/journal.pone.0240908 (PMC7575072; doi:10.1371/journal.pone.0240908)
Supplement: S7 Fig — (PDF) [file pone.0240908.s007.pdf]

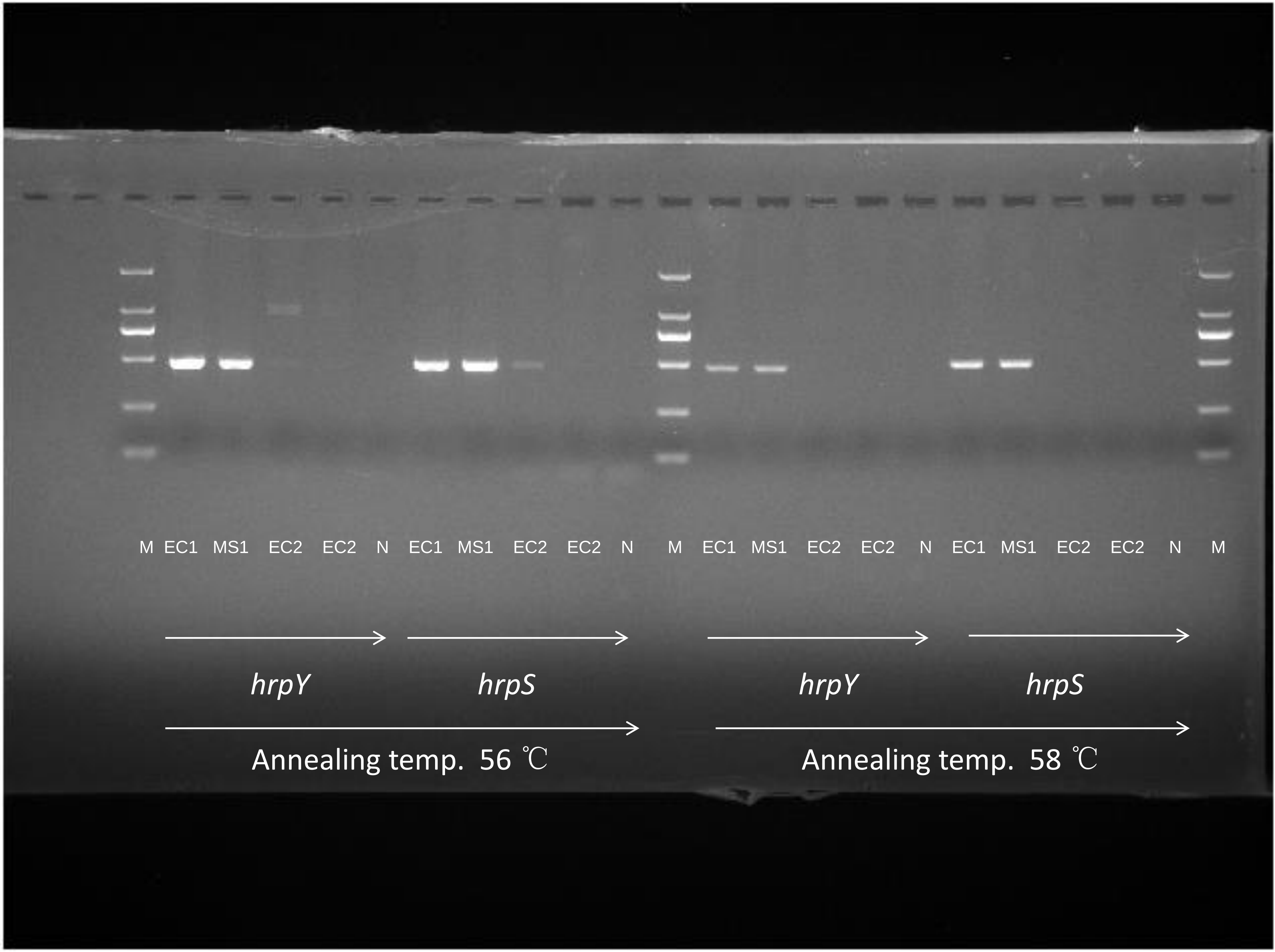
M EC1 MS1 EC2 EC2 N EC1 MS1 EC2 EC2 N M EC1 MS1 EC2 EC2 N EC1 MS1 EC2 EC2 N M
hrpY
hrpS
hrpY
hrpS
Annealing temp. 56 ℃
Annealing temp. 58 ℃

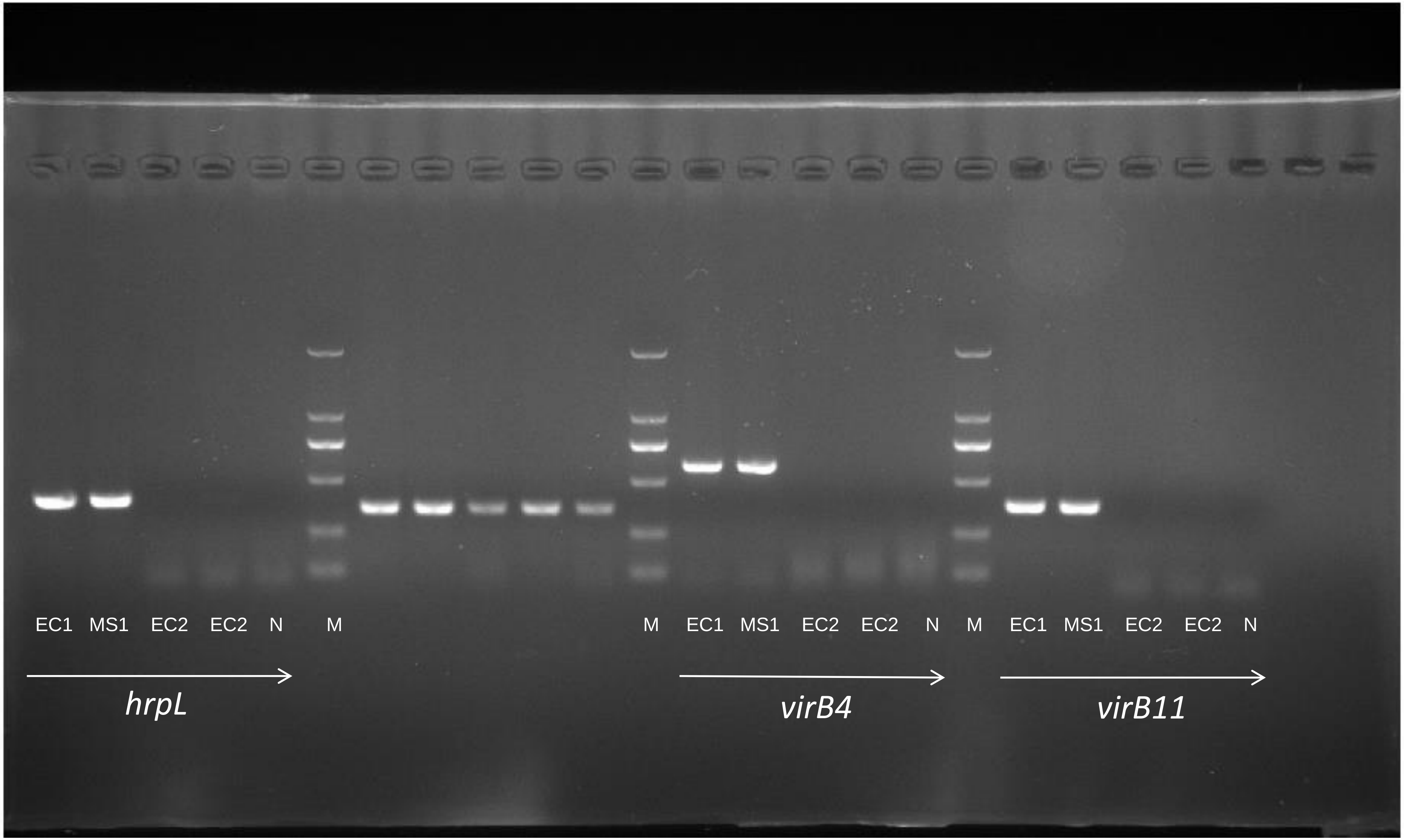
EC1 MS1 EC2 EC2 N M
hrpL
M EC1 MS1 EC2 EC2 N
virB4
M EC1 MS1 EC2 EC2 N
virB11

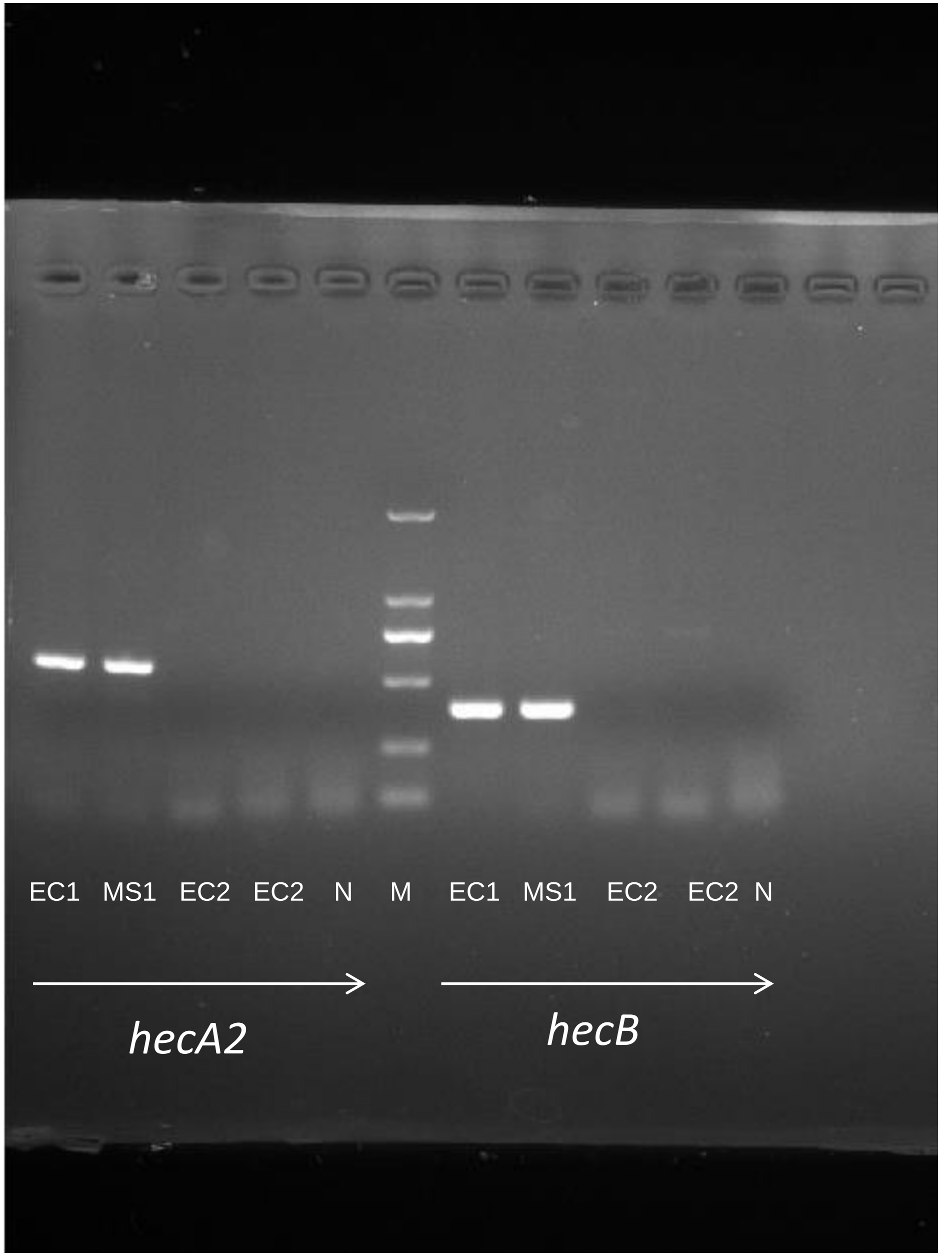
EC1 MS1 EC2 EC2 N M EC1 MS1 EC2 EC2 N
hecA2
hecB
